# Coupling of Physiological and Proteomic Analysis to Understand the Ethylene- and Chilling-Induced Kiwifruit Ripening Syndrome

**DOI:** 10.3389/fpls.2016.00120

**Published:** 2016-02-15

**Authors:** Ioannis S. Minas, Georgia Tanou, Evangelos Karagiannis, Maya Belghazi, Athanassios Molassiotis

**Affiliations:** ^1^Laboratory of Pomology, Department of Agriculture, Aristotle University of ThessalonikiThessaloniki, Greece; ^2^Department of Horticulture and Landscape Architecture, Colorado State UniversityFort Collins, CO, USA; ^3^Western Colorado Research Center at Orchard Mesa, Colorado State UniversityGrand Junction, CO, USA; ^4^UMR 7286 - CRN2M, Centre d' Analyses Protéomiques de Marseille, Centre National de la Recherche Scientifique, Aix-Marseille UniversitéMarseille, France

**Keywords:** *Actinidia deliciosa*, climacteric, mass spectrometry analysis, protein–protein interactions, proteomics, ripening, softening

## Abstract

Kiwifruit [*Actinidia deliciosa* (A. Chev.) C.F. Liang et A.R. Ferguson, cv. “Hayward”] is classified as climacteric fruit and the initiation of endogenous ethylene production following harvest is induced by exogenous ethylene or chilling exposure. To understand the biological basis of this “dilemma,” kiwifruit ripening responses were characterized at 20°C following treatments with exogenous ethylene (100 μL L^−1^, 20°C, 24 h) or/and chilling temperature (0°C, 10 days). All treatments elicited kiwifruit ripening and induced softening and endogenous ethylene biosynthesis, as determined by 1-aminocyclopropane-1-carboxylic acid (ACC) content and ACC synthase (ACS) and ACC oxidase (ACO) enzyme activities after 10 days of ripening at 20°C. Comparative proteomic analysis using two-dimensional gel electrophoresis (2DE-PAGE) and nanoscale liquid chromatography coupled to tandem mass spectrometry (nanoLC-MS/MS) revealed 81 kiwifruit proteins associated with ripening. Thirty-one kiwifruit proteins were identified as commonly regulated by the three treatments accompanied by dynamic changes of 10 proteins specific to exogenous ethylene, 2 to chilling treatment, and 12 to their combination. Ethylene and/or chilling-responsive proteins were mainly involved in disease/defense, energy, protein destination/storage, and cell structure/cell wall. Interactions between the identified proteins were demonstrated by bioinformatics analysis, allowing a more complete insight into biological pathways and molecular functions affected by ripening. The present approach provides a quantitative basis for understanding the ethylene- and chilling-induced kiwifruit ripening and climacteric fruit ripening in general.

## Introduction

Fruit ripening is a finely regulated developmental process that is orchestrated by the expression of ripening-related genes and proteins through a network of signaling pathways (Molassiotis et al., 2013). Fleshy fruit are largely divided into two groups on the basis of the presence (climacteric) or absence (non-climacteric) of increased respiration rate and biosynthesis of the plant hormone ethylene at the onset of ripening (Giovannoni, 2004). Nevertheless, members of the same species (e.g., tomato, melon, Japanese plums) have been reported to include both climacteric and non-climacteric varieties or mutations (Giovannoni, 2007; Minas et al., 2015). Ethylene biosynthesis occurs via a pathway involving two key biosynthetic enzymes: ACS, which converts *S*-adenosyl-L-methionine (SAM) into ACC, and ACO, which further converts ACC to ethylene (Yang and Hoffman, 1984). In climacteric fruit, ethylene is critical for the induction of fruit ripening since most of the ripening-related events are regulated by ethylene, whereas the non-climacteric fruit do not require ethylene for ripening (Zegzouti et al., 1999; Giovannoni, 2004; Zhang et al., 2012; Sorrequieta et al., 2013). There is also evidence for a chilling requirement to initiate ethylene-originated ripening in some climacteric fruit species. For example, some late pear cultivars, such as Passe Crassane, require a long chilling treatment (80 days, 0°C) before the fruit will be able to synthesize autocatalytically ethylene and ripen (El-Sharkawy et al., 2004). There is also strong evidence of ethylene and low temperature signaling interconnection, suggesting a chilling modulation of ethylene response. For instance, upstream receptor components of ethylene signaling can be induced by chilling treatment, as shown for *PcETR1a* expression in pear fruit (El-Sharkawy et al., 2003) and for *PpCTR1* and *PpEIN2* expression in peach fruit (Begheldo et al., 2008).

The ripening behavior of kiwifruit, which is classified into the climacteric group, is largely orchestrated by ethylene perception and biosynthesis (Kunsong et al., 1997; Antunes and Sfakiotakis, 2002; Antunes, 2007; Yin et al., 2008; Minas et al., 2012). Meanwhile, ‘Hayward’ kiwifruit also requires low temperature postharvest exposure (0°C) for the onset of ripening during the subsequent maintenance at room temperature (Antunes and Sfakiotakis, 2002). Studies on cold stored (0°C) “Sanuki Gold” kiwifruit (*Actinidia chinensis* Planch) have also demonstrated that low temperature modulates the ripening of kiwifruit in an ethylene-independent manner (Mworia et al., 2012). Despite these findings, no direct comparison between ethylene- and chilling-dependent ripening has been performed to examine differences and similarities in the molecular events involved in these processes. On this basis, the aim of this work was to investigate the impact of ethylene and chilling in kiwifruit ripening physiology. Kiwifruit proteins that were affected by ethylene and/or chilling during ripening were characterized using 2DE-nano LC-MS/MS based workflow. Particular attention was also paid to the prediction of the protein–protein interaction networks in ripened kiwifruit.

## Materials and methods

### Fruit material and experimental design

Kiwifruit (cv. “Hayward”), grown under standard cultural practices, were harvested from the experimental orchard of Aristotle University of Thessaloniki (Thessaloniki, Greece) at physiologically mature stage (mean weight: 93.1 ± 1.8 g, pericarp tissue firmness: 65.4 ± 1.4 N, core tissue firmness: 152.5 ± 4.6 N, soluble solids concentration (SSC): 6.4 ± 0.1%, titratable acidity: 1.9 ± 0.1%, dry weight: 16.3 ± 0.5%). Fruits were divided into 21 lots of 15 fruits each. One lot was analyzed at the time of harvest and the other lots (10+10) were left untreated or subjected to exogenous ethylene treatment (100 μL L^−1^) for 24 h at 20°C. The treatment with exogenous ethylene was performed in a stainless steel airtight tank (100 L) containing a vent for air circulation, while CO_2_ was absorbed with 500 mL of 4 M NaOH solution. At the end of the treatment, ethylene concentration in the tank was 108 μL L^−1^ while CO_2_ was 0.41%. Afterwards, untreated (control) and ethylene-treated (ethylene) fruit lots were split and half of them (5+5) kept at 20°C and their ripening behavior was analyzed 5, 10, 15, and 20 days after harvest (under non-chilling conditions). The other half of untreated and ethylene treated fruit lots (5+5) were transferred to cold storage (0°C, 90% RH, chilling conditions) for 10 days representing the chilling and ethylene and chilling treatments respectively, and then transferred to 20°C and their ripening behavior was determined following 0, 5, 10, 15, and 20 days upon removal from the chilling conditions. Overall, kiwifruits were subjected to four treatments (control, ethylene, chilling and ethylene and chilling), as described schematically in Supplementary Figure S1. It is noted that the experimental set concerning exogenous ethylene and chilling treatments was based on preliminary experiments.

During chilling storage and maintenance at the ripening room, ethylene was oxidized through KMnO_4_ filters (Purafil) and its levels were below the generally accepted levels for kiwifruit storage (10 nL L^−1^; data non shown). At each ripening day at 20°C (0, 5, 10, 15, or 20 days) following ethylene or following chilling treatment, ethylene production, respiration rate, pericarp and core tissue firmness, SSC and titratable acidity (TA), were monitored. Outer pericarp flesh samples were collected from each replication per sample (three batches of tissue from five fruits), flash frozen with liquid nitrogen and stored at −80°C until used for ethylene biosynthesis intermediates and enzymatic assays as well as for proteomic analysis.

### Physicochemical analysis of kiwifruit ripening behavior

Pericarp tissue firmness was measured by penetration at the two opposite cheeks of each fruit after peel (1 mm thick) removal using a fruit texture analyzer (model 53205, T.R. Turoni srl, Forlì, Italy) with a 8-mm probe, while core tissue firmness was measured by penetration, using the 8-mm probe, at the center of the two halves obtained from a transversal cut it in the equatorial region of each fruit. Data were recorded as Newtons (N) and firmness was expressed as the mean of 3 biological replications (five fruits per replication). SSC and TA were assessed in juice obtained from three biological replication of five fruits as elsewhere described (Minas et al., 2012). Statistical analysis performed using SPSS 19.0 for Mac OS X (SPSS, Chicago, IL, USA). Data (means consisted by three biological replications) were subjected to analysis of variance and least significant differences (LSD) at 5% level were used for means comparison.

### Ethylene production and respiration rate

For each treatment three replications of three fruits were weighed and placed into separate 2-L volume airtight jars for 30 min. Ethylene production was measured by withdrawing a 1-mL headspace gas sample from each jar and injecting it into a gas chromatograph (model 3300, Varian Analytical Instruments, CA, USA), equipped with a stainless steel column filled with Porapak (length 100 cm, diameter 0.32 cm) and a flame ionization detector (Minas et al., 2012). Respiration rate (RR) was calculated by CO_2_ concentration in the gas phase of the jars, determined by withdrawing an 1-mL headspace gas sample from each jar and injecting it into an infrared gas analyzer (Combo 280, David Bishop Instruments, UK; Minas et al., 2012). Statistical analysis performed as described above (Section Physicochemical Analysis of Kiwifruit Ripening Behavior).

### Analysis of ethylene biosynthesis intermediates and enzyme activities

1-aminocyclopropane-1-carboxylic acid (ACC) and 1-malonyl-aminocyclo-propane-1-carboxylic acid (MACC) contents as well as of ACC synthase (ACS) and ACC oxidase (ACO) enzyme activities were analyzed as previously described (Bulens et al., 2011). Statistical analysis performed as described above (Section Physicochemical Analysis of Kiwifruit Ripening Behavior).

### Two-dimensional gel electrophoresis, image acquisition, and analysis

Fruit pericarp flesh was ground in liquid nitrogen and soluble proteins were extracted as previously described (Tanou et al., 2012). Protein concentration was determined following Bradford's method (Bradford, 1976), using BSA as standard. Proteins (50 μg) were separated by isoelectrofocusing on 3-10 NL IPG strips (11 cm; Biorad). The second dimension was carried out at 12.5% Tris-HCl polyacrylamide gels (Biorad). Three gels representing three biological replicates were run in parallel for each treatment and stained with silver nitrate. 2DE-gels were scanned with Bio-Rad GS-800 Calibrated Densitometer and analyzed with PDQuest Advanced 2-D Gel Analysis software (version 8.1, Bio-Rad) as previously described (Tanou et al., 2009). Statistical analysis was done by one-way analysis of variance (*P* < 0.05) and individual means were compared using Student's *t*-test (significance level 95%). The statistical significant differences were further combined by the quantitative two-fold change of spot volume (Supplementary Table S1).

### Mass spectrometry analysis

Gels stained with the Silver stain plus kit (Biorad) and selected spots were analyzed by LTQ-Velos-Orbitrap online with a nanoLC Ultimate 3000 chromatography system (Thermo Fisher Scientific, Bremen, Germany). For protein identification, MS/MS experiments were performed as elsewhere reported (Vu Hai et al., 2013). Searches were done against the Cornell University kiwifruit protein database (http://bioinfo.bti.cornell.edu/cgi-bin/kiwi/download.cgi) containing 39,004 protein sequences and National Center for Biotechnology Information (NCBI) databases using BLASTp analyses using MASCOT software. Significant differences were analyzed through the two-way hierarchical clustering using Permut Matrix software. The row-by-row normalization of data was performed using zero-mean and unit-standard deviation technique. Pearson's distance and Ward's algorithm were used for the analysis. Among the positive matches, protein identifications based on at least two different peptide sequences of more than six amino acids with an individual score above 20 were accepted (identity peptide scores corresponding to *P* < 0.05 was 18 for search in KIWIFRUIT GENOME); in some cases, the protein sequences obtained were BLASTed manually against the current databases. When presented identifications were based on single peptide, the identity score was required and additional information provided. All peptide sequences, accession numbers, database source, matching criteria, Mascot scores, and sequence coverage are given in Supplementary Table S2.

### Bioinfomatic analysis

To understand functions and interactions of identified proteins, a protein–protein interaction network (PPI) was predicted with the online analysis tool STRING 9.0 (http://string-db.org; Szklarczyk et al., 2011). Since protein identification was based upon different organisms listed in the National Center for Biotechnology Information database (NCBI) Viridiplantae and the Kiwifruit Genome database, all identified proteins were blasted against the *Arabidopsis thaliana* TAIR10 (The Arabidopsis Information Resource) protein database (http://www.arabidopsis.org/) with the intention of obtaining annotated protein entries for PPI tools. Results with the highest score and lowest *E*-value were considered as relevant for each identified protein (Supplementary Table S3). Biological processes and molecular functions of PPI were predicted by Biological Networks Gene Ontology tool (BiNGO, 2.44; Maere et al., 2005), a plugin for Cytoscape. A hypergeometric test with Benjamini and Hochberg correction, *P*-value of 0.001 and *A. thaliana* taxonomy were selected for search parameters.

## Results

### Physiological responses of kiwifruit challenged with exogenous ethylene and chilling

**Figure 1:**
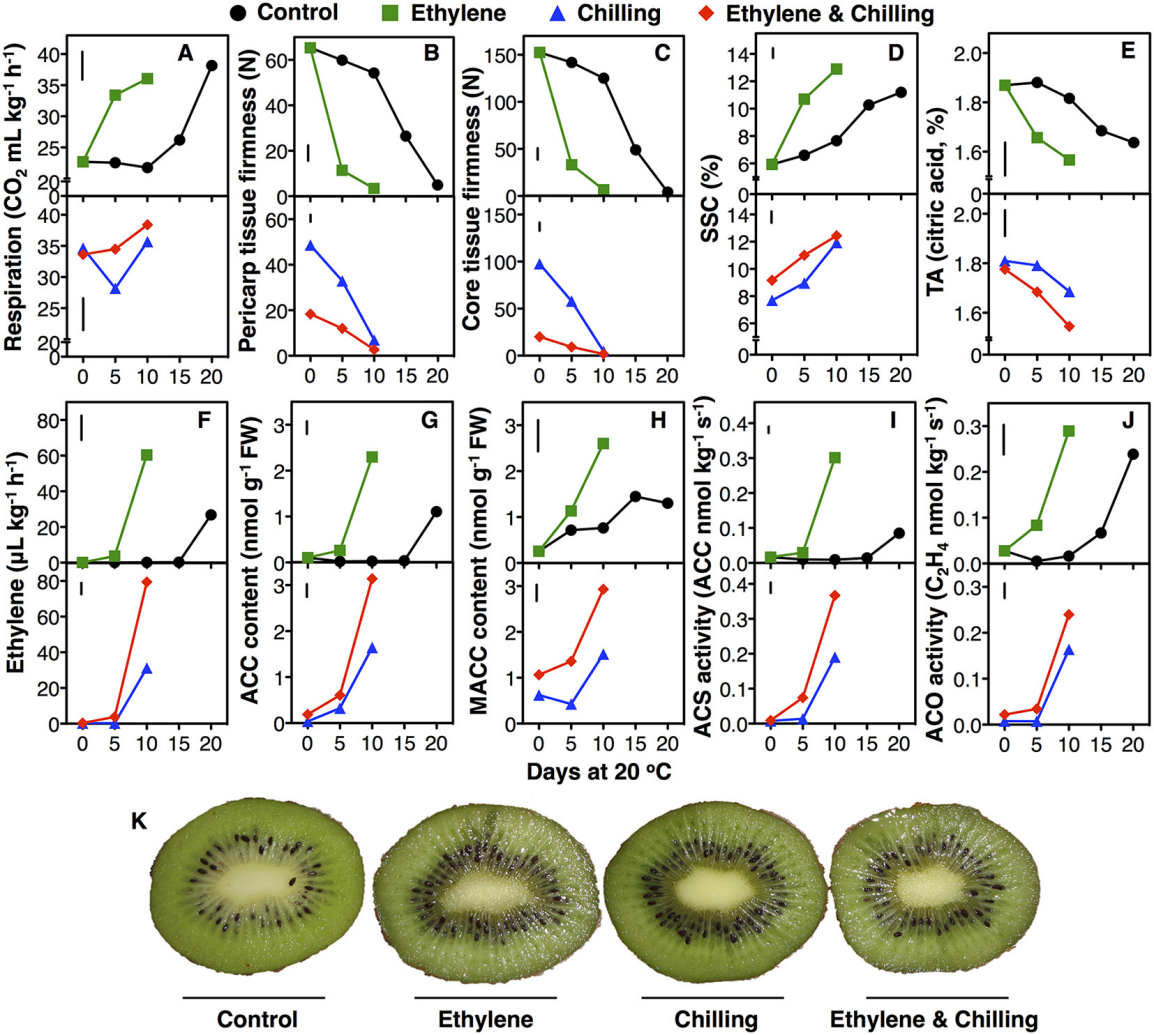
**Kiwifruit ripening and endogenous ethylene biosynthesis was induced by exogenous ethylene and chilling treatments**. Following harvest kiwifruit (cv. “Hayward”) were treated with exogenous ethylene (100 μL^−1^, 20°C, 24 h) or not (control) and then left at 20°C or cold stored (0°C, RH 90%, 10 days) and subsequently transferred at 20°C to characterize their ripening performance. Changes of respiration rate **(A)**, firmness of outer pericarp **(B)** and core tissue **(C)**, soluble solids concentration (SSC, **D**), titratable acidity (TA, **E**), ethylene production **(F)**, content of ACC **(G)**, and MACC **(H)**, and enzymatic activities of ACS **(I)** and ACO **(J)** in kiwifruit during ripening at 20°C. Phenotypes of kiwifruit at 10 days of ripening at 20°C following harvest (non-chilling) or chilling exposure **(K)**. Each value represents the mean of three biological replications of five fruits analyzed at each ripening stage. The vertical bar in each particular figure plate represents the least significant difference (LSD, *P* = 0.05), which was used for means comparison between the different treatments and ripening times.

In the absence of chilling conditions (herein designed as “non-chilling conditions”), exogenous ethylene application immediately after harvest induced kiwifruit ripening, as documented by the increase (48%) in respiration rate (RR) compared with control fruit after 5 days of ripening at 20°C, while the peak of RR in ethylene-treated fruit was observed after 10 days of ripening (Figure 1A). Moreover, the initiation of respiration climacteric (16% increase compared with harvest) in control fruit was recorded after 15 days. In contrast, fruit exposed to low temperature (0°C; herein designed as “chilling conditions”) for 10 days after harvest (chilling treatment or ethylene and chilling treatment) exhibited an increase in RR by 50% following removal from chilling conditions (0 days at 20°C). Afterwards, chilling-treated fruit showed an 18% reduction of RR after 5 days ripening at 20°C, while RR increased again following 10 days to the same levels as recorded at 0 day. On the other hand, RR was continuously increased during ripening at 20°C for up to 10 days in kiwifruit exposed to ethylene and chilling treatment.

Ethylene-treated kiwifruit that ripened for 5 days at 20°C exhibited a rapid softening of pericarp and core tissue by 83 and 92%, respectively; afterwards fruit softening continued leading to low pericarp and core tissue firmness after 10 days ripening at 20°C (3.5 and 2.8 N, respectively, Figures 1B,C). In contrast, control fruit softened with a markedly slower rate since the pericarp and core tissue firmness were reduced only by 18% after 10 days of ripening at 20°C, while after 20 days the pericarp and the core tissue firmness were reduced to 4.9 and 4.1 N, respectively. Compared with their initial pericarp and core tissue firmness at harvest, the chilling-treated fruit lost 25 and 36% of their initial firmness during the 10-days cold storage period, while ethylene and chilling-treated fruit lost 72 and 87% of their firmness, respectively. Fruit that exposed only to chilling displayed 6.2 and 6.6 N firmness following 10 days ripening, while ethylene and chilling-treated fruit had 2.7 and 1.8 N pericarp and core tissue firmness, respectively. SSC of ethylene-treated kiwifruit was increased rapidly by 80% the first 5 days of ripening, while the highest value observed after 10 days. Control fruit exhibited only a 30% increase in SSC after 10 days of ripening, while the highest value was recorded after 20 days (Figure 1D). Upon transfer at room temperature (0 days at 20°C), chilling treatments caused an increase in SSC by 30 and 55% in chilling-treated as well as in ethylene and chilling-treated fruit, respectively; the highest SSC values observed after 10 days of ripening in both treatments (11.9 and 12.4% for chilling and ethylene and chilling-treated fruit, respectively, Figure 1D). Although titratable acidity (TA) remained unchanged during ripening in control fruit, ethylene treatment caused a reduction of TA by 11% at 5 days, while the lowest TA value in ethylene-treated fruit recorded after 10 days of ripening (Figure 1E). TA remained unaffected upon removal from chilling conditions (0 day at 20°C) but it was reduced during ripening for 10 days by 10 and 18% in chilling and in ethylene and chilling-treated kiwifruit, respectively. It is noted that fruit subjected to postharvest ethylene and chilling treatments were phenotypically distinct from control fruit after 10 days ripening (Figure 1K), testifying the above physiological data.

### Induction of ethylene biosynthesis by exogenous ethylene and chilling treatment

The initiation of endogenous ethylene production in fruit exposed to exogenous ethylene was observed after 5 days of ripening at 20°C, while the peak of ethylene production was detected at 10 days. Control fruit didn't exhibit any ethylene production prior to 15 days of ripening at 20°C and also showed lower levels of ethylene production than ethylene-treated fruit (Figure 1F). Chilling also increased ethylene production after 10 days of ripening. In ethylene and chilling-treated fruit ethylene production initiated after 5 days at 20°C, while the peak of ethylene production was occurred after 10 days of ripening.

Since ethylene pattern was considerably affected by exogenous ethylene and/or chilling treatment, intermediates content (ACC and MACC) and enzymatic activities (ACS and ACO) of ethylene biosynthesis pathway were analyzed. Exogenous ethylene, in the absence of chilling, provoked ACC accumulation after 5 days of ripening, while the highest ACC level was recorded after 10 days of ripening (Figure 1G). In contrast, ACC content was increased in control fruit only after 20 days of ripening, although it was 50% lower compared with the peak observed in ethylene-treated fruit. Chilling treatment also induced ACC accumulation after 5 days of ripening; the highest ACC level was observed after 10 days (1.6 and 3.1 nmol kg s^−1^ FW for chilling- and ethylene and chilling-treated, respectively). Meanwhile, MACC content was increased after 5 days ripening in ethylene-treated fruit and the highest MACC value was recorded after 10 days (2.6 nmol kg^−1^ FW; Figure 1H); however, MACC accumulation was lower in control fruit and the highest value observed after 15 days (1.4 nmol kg^−1^ FW). Ethylene and chilling-treated kiwifruit showed greater MACC accumulation compared to chilling-treated kiwifruit during the ripening period.

Exogenous ethylene treatment enhanced ACS activity following 10 days of ripening (0.3 nmol ACC kg s^−1^) compared with control fruit which showed undetectable levels of ACS activity after 15 days of ripening and only a slight increase after 20 days (<0.1 nmol ACC kg s^−1^; Figure 1I). In fruit treated with ethylene and chilling, an increase in ACS activity was recorded after 5 days of ripening and the highest value recorded after 10 days (0.37 nmol ACC kg s^−1^). ACS activity was increased after 10 days of ripening following chilling application, however was lower compared to ethylene and chilling-treated fruit (0.19 nmol ACC kg s^−1^). Meanwhile, ACO activity following harvest was increased after 5 and 15 days of ripening in ethylene-treated and control fruit, respectively, while the highest ACO activity levels were observed after 10 days (0.29 nmol C_2_H_4_ kg s^−1^) and 20 days (0.24 nmol C_2_H_4_ kg s^−1^), respectively (Figure 1J). Chilling exposure in ethylene-treated and untreated fruit resulted in increased ACO activity after 10 days of ripening.

### Abundance changes and functional classification of the ethylene- and chilling-responsive kiwifruit proteins

**Figure 2:**
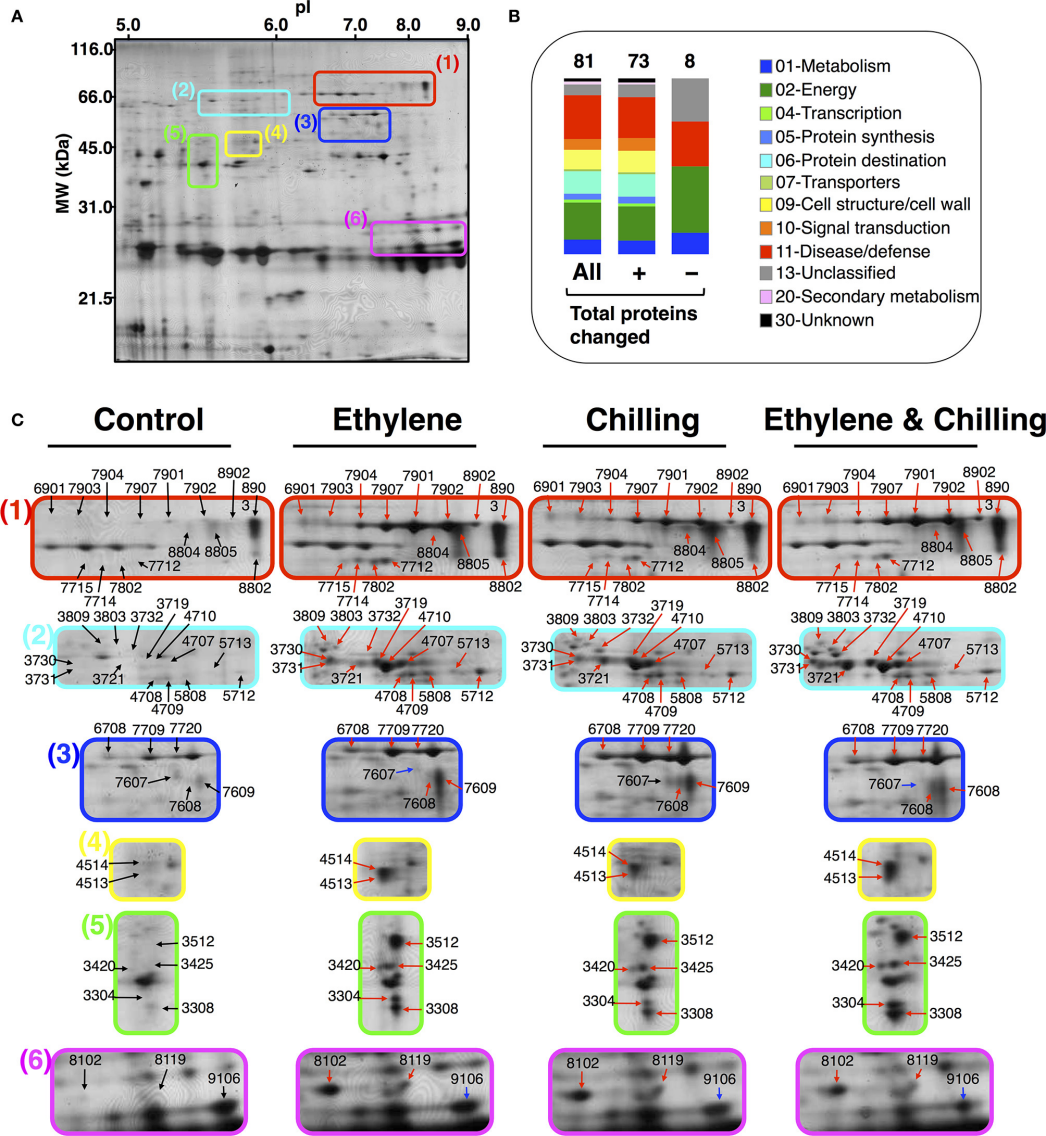
**2-DE proteome maps of kiwifruit (cv. “Hayward”) following ethylene and chilling treatments. (A)** Representative protein spots of pericarp flesh tissue of control kiwifruit. Equal amount (50 μg) of total protein extracts was loaded in each gel. **(B)** Functional categories of identified kiwifruit proteins represented with color code. **(C)** Close up areas of the 2DE-gels showing variation in the protein spots intensity as shown in **(A)** for control and for kiwifruit exposed to ethylene, chilling and ethylene and chilling. For each treatment, 2DE-maps were run in triplicate and for a minimum of three independent extractions. Identified protein spots correspond to those marked in Table 1. Black, red, and blue arrows indicate protein spots that were unchanged, increased, or decreased in abundance, respectively, in kiwifruit exposed to ethylene and/or chilling treatments compared with control fruit. (+) and (−) symbols indicate identified kiwifruit proteins that were increased or decreased in abundance, respectively.

A proteomic analysis was performed to identify changes in kiwifruit proteins that were affected by the different postharvest conditions. Based on the physiological data (Figure 1), samples were collected from the pericarp tissue of the fruits that were ripened for 10 days at 20°C following exposure to different experimental conditions. It should be mentioned that kiwifruit protein identification was an analytical challenge because of the lack of complete genome sequence information for *Actinidia* spp. More than 270 protein spots were detected using PDQuest Advanced 2-D Gel Analysis software following the 2-DE gel analysis. On the basis of the image and statistical analyses using Student's *t*-test further validated by the two-fold change threshold, a total of 73 protein spots were associated with significant volume changes in kiwifruit exposed to postharvest treatments. Protein spots of interest were excised, trypsin-digested, analyzed by nanoLC/MS/MS, and then the mass spectrum was conducted by searching the Cornell University kiwifruit protein database and the NCBI non-redundant green plant database using the MASCOT tools (Matrix Science). From the mass spectrometric analysis, 67 of the 73 spots were matched to be peptide signals and, among them, 65 spots were successfully matched to proteins in either the NCBInr or kiwifruit genome database by MASCOT software. Following this approach, 81 kiwifruit proteins were fully identified (Figures 2A, B). Thirteen proteins were identified in more than one spot, indicating that a number of the differentially expressed spots were either subjected to post-translational modification or were members of multigenic protein families. Among these multi-spot identified proteins were β-D-galactosidase (Figure 2C plate 1, spots: 7907, 7902, 8804, 7901, 7903, 7904, 8902), remorin (Figure 2C plate 1, spots: 7715, 7802, 8802), viral-A-type inclusion protein repeat containing protein expressed (Figure 2C plate 2, spots: 3719, 3721), thaumatin (Figure 2C plate 6, spots: 8102, 9106) and natterin (Figure 2C plate 3, spots: 7608, 7709, 6708, 7720, 7607).

To generate an overview of the most relevant biological processes involved in kiwifruit postharvest ripening, a categorization of differentially accumulated proteins was performed as described (Bevan et al., 1998) and presented in Figure 2B. Seventy-three of the identified kiwifruit proteins were already known proteins and functionally characterized, while two were characterized as unknown proteins and 6 were not characterized. The identified kiwifruit proteins were mainly related to disease/defense (24.7%) following by energy (21.0%), protein destination/storage (12.4%) and cell structure/cell wall (11.1%). A complete list of the protein identification, including also peptide sequences, accession number, subcellular localization and matching criteria is presented in Table 1; Supplementary Table S2. Based on database searches, identified proteins that were predicted to be localized in the cytoplasm, extracellular, chloroplast and cell wall were the largest groups accounting for 23, 19, 17, and 12% of all identified proteins, respectively. From the taxonomic viewpoint, among the 81 presently identified kiwifruit proteins, 33 matched to available kiwifruit sequences of the *Actinidia* spp., 11 belonged to *Ricinus* spp., 9 belonged to *Vitis* spp., 1 to *Prunus* spp. and the remaining 29 were associated with other green plant species.

**Table 1:** **Protein identification of kiwifruit pericarp flesh following ethylene and chilling treatments by 2DE-PAGE and mass spectrometry LC/MS-MS**.

**Spot No^a^**	**Protein name^b^**	**Accession number^c^**	**Ethylene^d^**	**Chilling^e^**	**Ethylene and Chilling^f^**	**Ethylene and Chilling vs. Ethylene^g^**	**Subcellular localization^i^**	**Organism^j^**	**Functional categories^k^**	**Th. pI/Mw^l^**
2104	Thaumatin	gi|190358875	**—**^h^	**—**	↑	↑	Extracellular	*Actinidia deliciosa*	11.02-Disease/defense/Defense-related	8.29/24205
2304	Kiwellin	gi|441482352	**—**	**—**	↑	**—**	Extracellular	*Actinidia deliciosa*	11.02-Disease/defense/Defense-related	5.28/22228
2402	Malate dehydrogenase	Achn121571	**—**	↓	**—**	**—**	Chloroplast	*Nicotiana tabacum*	02.30-Energy/Photosynthesis	7.59/43574
2509	Protease C56	Achn264591	**—**	**—**	**—**	↑	Chloroplast	*Ricinus communis*	06.13-Protein destination and storage/Proteolysis	6.01/44271
2711	Chaperonin CPN60	gi|356534856	↑	↑	↑	**—**	Mitochondrion	*Glycine max*	06.01-Protein destination and storage/Folding and stability	5.99/61052
2716	Leucine aminopeptidase	Achn369241	↑	↑	↑	**—**	Chloroplast	*Ricinus communis*	06.13-Protein destination and storage/Proteolysis	7.59/34132
3206	Abscisic stress ripening protein	Achn305291	**—**	**—**	↑	↑	Nucleus	*Prunus persica*	11.05-Disease/Defense/Stress responses	5.88/20048
3304	Glutelin type-A	gi|195225381	↑	↑	↑	↑	Cytoplasm	*Actinidia chinensis*	06.20-Protein destination and storage/Storage proteins	5.29/19669
3307	Glutelin type-A	Achn231381	↑	**—**	↑	**—**	Cytoplasm	*Actinidia chinensis*	06.20-Protein destination and storage/Storage proteins	6.85/37197
3308	Glutelin type-A	gi|195248852	↑	↑	↑	**—**	Cytoplasm	*Actinidia deliciosa*	06.20-Protein destination and storage/Storage proteins	5.55/22101
3417	Lactoylglutathione lyase	Achn326191	**—**	**—**	**—**	↑	Vacuole	*Gossypium hirsutum*	11.06-Disease/Defense/Detoxification	4.92/33647
3420	ATP synthase subunit C	Achn017021	**—**	**—**	**—**	↑	Vacuole	*Ricinus communis*	07.22-Transporters/Transport ATPases	6.13/50208
3425	Malate dehydrogenase	Achn221601	↑	↑	↑	**—**	Mitochondrion	*Glycine max*	02.10-Energy/TCA pathway	8.66/35926
3425	Polyphenoloxidase	Achn226891	↑	↑	↑	**—**	Chloroplast	*Camellia nitidissima*	20.1-Secondary metabolism/Phenylpropanoids/Phenolics	6.27/65115
3504	Kiwellin	gi|85701136	↑	↑	↑	**—**	Extracellular	*Actinidia deliciosa*	11.02-Disease/defense/Defense-related	5.83/19955
3505	Kiwellin	gi|85701136	**—**	↑	↑	↑	Extracellular	*Actinidia deliciosa*	11.02-Disease/defense/Defense-related	5.83/19955
3512	Enolase	gi|14423687	↑	↑	↑	**—**	Cytoplasm	*Hevea brasiliensis*	02.01-Energy/Glycolysis	5.92/47884
3622	Enolase	Achn354501	↑	↑	**—**	**—**	Cytoplasm	*Vitis vinifera*	02.01-Energy/Glycolysis	5.80/61843
3622	Asparaginyl-tRNA synthetase	Achn069631	↑		**—**	**—**	Chloroplast	*Vitis vinifera*	05.10-Protein synthesis/tRNA synthases	7.67/72462
3622	Phosphoenolpyruvate carboxylase	Achn133831	↑	↑	**—**	**—**	Cytoplasm	*Gossypium hirsutum*	02.02-Energy/Gluconeogenesis	5.77/110449
3719	Viral A-type inclusion protein repeat containing protein expressed	gi|356510118	↑	**—**	↑	**—**	Nucleus	*Glycine max*	10.04-Signal transduction	5.01/108732
3721	Viral A-type inclusion protein repeat containing protein expressed	gi|356510118	↑	↑	↑	↑	Nucleus	*Glycine max*	10.04-Signal transduction	5.01/108732
3730	D-3-phosphoglycerate dehydrogenase	Achn006391	↑	**—**	**—**	**—**	Chloroplast	*Vitis vinifera*	01.01-Metabolism/Amino Acid	6.27/ 62136
3731	Glucan endo-1,3-beta-glucosidase	Achn236981	↑	**—**	↑	**—**	Vacuole	*Ricinus communis*	11.02-Disease/defense/Defense-related	5.92/ 51329
3732	Pyruvate decarboxylase	Achn036401	**—**	**—**	↑	↑	Cytoplasm	*Glycine max*	11.05-Disease/Defense/Stress responses	5.96/ 67889
3732	HSP70 luminal binding	Achn177881	**—**	**—**	↑	↑	Endoplasmic reticulum	*Theobroma cacao*	06.01-Protein destination and storage/Folding and stability	5.03/ 70395
3803	Non-identified		↑	↑	↑	**—**			13-Unclassified	
3809	Beta-glucosidase	Achn262021	↑	**—**	↑	**—**	Cytoplasm	*Vitis vinifera*	01.05-Metabolism/Sugars and polysaccharides	8.15/82399
3809	Alpha-mannosidase	Achn348701	↑	**—**	↑	**—**	Lysosome	*Ricinus communis*	06.07-Protein destination and storage/Modification	7.18/182303
4003	Bet v 1 related allergen	gi|281552896	**—**	**—**	↑	**—**	Cytoplasm	*Actinidia chinensis*	11.02-Disease/defense/Defense-related	5.82/17479
4512	Enolase	Achn086741	↑	↑	**—**	**—**	Cytoplasm	*Vitis vinifera*	02.01-Energy/Glycolysis	5.20/45005
4512	Aspartate aminotransferase	Achn186891	↑	↑	**—**	**—**	Chloroplast	*Vitis vinifera*	01.01-Metabolism/Amino acid	6.68/45730
4513	Monodehydroascorbate reductase	gi|284437984	↑	↑	↑	**—**	Cytoplasm	*Actinidia deliciosa*	11.06-Disease/Defense/Detoxification	7.22/25481
4514	Enolase	Achn354501	**—**	↑	↑	**—**	Cytoplasm	*Vitis vinifera*	02.01-Energy/Glycolysis	5.80/61843
4707	Phosphoenolpyruvate carboxykinase	gi|195203610	↑	↑	↑	**—**	Cytoplasm	*Actinidia chinensis*	02.02-Energy/Gluconeogenesis	5.42/17394
4708	Mitochondrial-processing peptidase	Achn043531	↑	**—**	**—**	**—**	Mitochondrion	*Vitis vinifera*	06.04-Protein destination and storage/Targeting	6.30/61126
4709	Delta-1-pyrroline-5-carboxylate dehydrogenase	gi|149938952	↑	**—**	↑	**—**	Mitochondrion	*Actinidia chinensis*	01.01-Metabolism/Amino acid	6.36/62007
4710	Pyruvate decarboxylase	gi|51587336	↑	↑	↑	**—**	Chloroplast	*Lotus japonicus*	11.05-Disease/Defense/Stress responses	5.76/62637
5405	Transketolase	gi|195315378	↑	**—**	**—**	↓	Chloroplast	*Actinidia chinensis*	02.30-Energy/Photosynthesis	7.21/22047
5407	GDP-mannose 4,6-dehydratase	Achn053211	**—**	**—**	**—**	↓	Mitochondrion	*Nicotiana benthamiana*	01.05-Metabolism/Sugars and polysaccharides	6.24/42180
5712	Non-identified		↑	**—**	**—**	↓			13-Unclassified	
5713	Phosphoenolpyruvate carboxykinase	Achn041831	↑	**—**	**—**	**—**	Cytoplasm	*Ricinus communis*	02.02-Energy/Gluconeogenesis	7.12/78671
5808	Pyruvate decarboxylase	Achn219321	↑	**—**	↑	**—**	Cytoplasm	*Citrus sinensis*	11.05-Disease/Defense/Stress responses	6.16/63990
5907	Alpha-glucosidase	Achn221901	**—**	↑	**—**	**—**	Cytoplasm	*Ricinus communis*	01.05-Metabolism/Sugars and polysaccharides	6.24/97449
5909	Eukaryotic translation elongation factor	Achn004851	↑	↑	↑	**—**	Ribosome	*Ricinus communis*	05.04-Protein synthesis/Translation factors	6.30/117237
5921	Uncharacterized protein 2	Achn042731	**—**	**—**	↑	**—**	Nucleus	*Vitis vinifera*	30-Unknown	4.68/123420
6509	Non-identified		↑	**—**	↑	↓			13-Unclassified	
6604	UDP-glucose dehydrogenase	Achn256641	↑	**—**	**—**	**—**	Chloroplast	*Nicotiana tabacum*	01.05-Metabolism/Sugars and polysaccharides	5.72/39637
6614	Non-identified		↑	**—**	↑	**—**			13-Unclassified	
6708	Natterin	gi|195285924	**—**	**—**	↑	**—**	Extracellular	*Actinidia deliciosa*	11.02-Disease/defense/Defense-related	8.97/25502
6901	Non-identified		↑	↑	↑	**—**			13-Unclassified	
6946	Kiwellin	Achn107521	↑	**—**	**—**	**—**	Extracellular	*Actinidia deliciosa*	11.02-Disease/defense/Defense-related	4.72/22356
7211	Actinidin	gi|195214977	↑	↑	↑	**—**	Extracellular	*Actinidia deliciosa*	06.13-Protein destination and storage/Proteolysis	5.45/19222
7212	2-oxoglutarate dehydrogenase	Achn007391	**—**	↑	↑	↑	Mitochondrion	*Ricinus communis*	02.10-Energy/TCA pathway	5.75/102017
7607	Natterin	gi|195285924	↑	**—**	**—**	**—**	Extracellular	*Actinidia deliciosa*	11.02-Disease/defense/Defense-related	8.97/25502
7607	NADP-dependent malic enzyme	Achn312431	↓	**—**	**—**	**—**	Chloroplast	*Ricinus communis*	02.10-Energy/TCA pathway	7.16/57669
7608	Natterin	Achn294821	**—**	**—**	↑	**—**	Extracellular	*Thalassophryne nattereri*	11.02-Disease/defense/Defense-related	6.61/52945
7609	Pectinesterase	gi|160419153	↑	**—**	↑	**—**	Cell wall	*Actinidia deliciosa*	09.01-Cell structure/Cell wall	6.67/35346
7709	Natterin	gi|195285924	↑	**—**	↑	**—**	Extracellular	*Actinidia deliciosa*	11.02-Disease/defense/Defense-related	8.97/25502
7712	Non-identified		↑	↑	↑	**—**			13-Unclassified	
7714	NADP-dependent malic enzyme	Achn312431	↑	**—**	↑	**—**	Chloroplast	*Ricinus communis*	02.10-Energy/TCA pathway	7.16/57669
7715	Remorin	Achn227731	**—**	**—**	↑	**—**	Nucleus	*Medicago truncatula*	10.04-Signal transduction	9.92/64873
7720	Natterin	Achn294821	**—**	**—**	↑	**—**	Extracellular	*Thalassophryne nattereri*	11.02-Disease/defense/Defense-related	6.61/52945
7802	Remorin	Achn227731				**—**	Nucleus	*Medicago truncatula*	10.04-Signal transduction	9.92/64873
7901	Beta-D-galactosidase	gi|318136780	↑	↑	↑	**—**	Cell wall	*Actinidia deliciosa*	09.01-Cell structure/Cell wall	5.71/81070
7902	Beta-D-galactosidase	gi|318136780	↑	↑	↑	**—**	Cell wall	*Actinidia deliciosa*	09.01-Cell structure/Cell wall	7.51/81070
7903	Beta-D-galactosidase	gi|318136780	↑	↑	↑	**—**	Cell wall	*Actinidia deliciosa*	09.01-Cell structure/Cell wall	7.51/81070
7904	Beta-D-galactosidase	gi|318136780	↑	↑	↑	**—**	Cell wall	*Actinidia deliciosa*	09.01-Cell structure/Cell wall	7.51/81070
7907	Beta-D-galactosidase	gi|318136780	↑	↑	↑	**—**	Cell wall	*Actinidia deliciosa*	09.01-Cell structure/Cell wall	7.51/81070
8102	Thaumatin	gi|146737976	↑	↑	↑	**—**	Extracellular	*Actinidia deliciosa*	11.02-Disease/defense/Defense-related	7.92/21614
8119	Formate dehydrogenase	gi|195211331	↑	↑	↑	**—**	Mitochondrion	*Actinidia deliciosa*	11.06-Disease/Defense/Detoxification	9.02/17186
8119	Catalase	Achn051741	↑	↑	↑	**—**	Peroxisome	*Gossypium hirsutum*	11.06-Disease/Defense/Detoxification	6.74/52770
8415	Uncharacterized protein 3	gi|195193220	↑	↑	**—**	**—**	Chloroplast	*Actinidia deliciosa*	30-Unknown	8.01/13873
8604	RNase Phy3, partial	gi|195320811	↑	↑	↑	**—**	Extracellular	*Actinidia deliciosa*	04.99-Transcription/Others	8.27/22260
8605	Elongation factor	gi|61741088	↑	↑	**—**	**—**	Cytoplasm	*Actinidia deliciosa*	05.04-Protein synthesis/Translation factors	9.15/49233
8802	Remorin	Achn227731	↑	**—**	**—**	**—**	Nucleus	*Medicago truncatula*	10.04-Signal transduction	9.92/64873
8804	Beta-D-galactosidase	gi|318136780	↑	↑	↑	**—**	Cell wall	*Actinidia deliciosa*	09.01-Cell structure/Cell wall	7.51/81070
8805	Quinohemoprotein ethanol dehydrogenase	gi|195257582	↑	↑	↑	**—**	Cell wall	*Actinidia deliciosa*	02.20-Energy/Electron-transport	9.63/16255
8902	Beta-D-galactosidase	gi|318136780	↑	↑	↑	**—**	Cell wall	*Actinidia deliciosa*	09.01-Cell structure/Cell wall	7.51/81070
8903	Quinohemoprotein ethanol dehydrogenase	gi|195196846	↑	↑	↑	**—**	Nucleus	*Actinidia deliciosa*	02.20-Energy/Electron-transport	5.31/21355
9106	Thaumatin	gi|190358875	**—**	**—**	↓	**—**	Extracellular	*Actinidia deliciosa*	11.02-Disease/defense/Defense-related	8.29/24205

a *Spot No, spot label on the reference 2DE-gel maps*;

b *Protein name, identified peptide names*;

c *Acces. number, accession number in NCBI or Kiwifruit Genome database*;

d *Ethylene, protein whose accumulation status was changes by Ethylene treatment in comparison to Control*;

e *Chilling, protein whose accumulation status was changes by Chilling treatment in comparison to Control*;

f *Ethylene and Chilling, protein whose accumulation status was changes by combined Ethylene and Chilling treatment in comparison to Control*;

g *Ethylene and Chilling vs. Ethylene, protein whose accumulation status was changes by combined Ethylene and Chilling treatment in comparison to of Ethylene alone treatment*;

h *Responsiveness to treatment pattern of accumulation; —, protein whose accumulation level was constant; ↑, increased abudance protein; ↓, decreased abudance protein*;

i *Subcellular localization, Sub-cellular if identified proteins*;

j *Organism, organism in which the protein has been identified*;

k *Functional category, classification of identified proteins into functional categories*;

l *Theor. pI/Mw, theoretical isoelectric point/molecular weight*.

**Figure 3:**
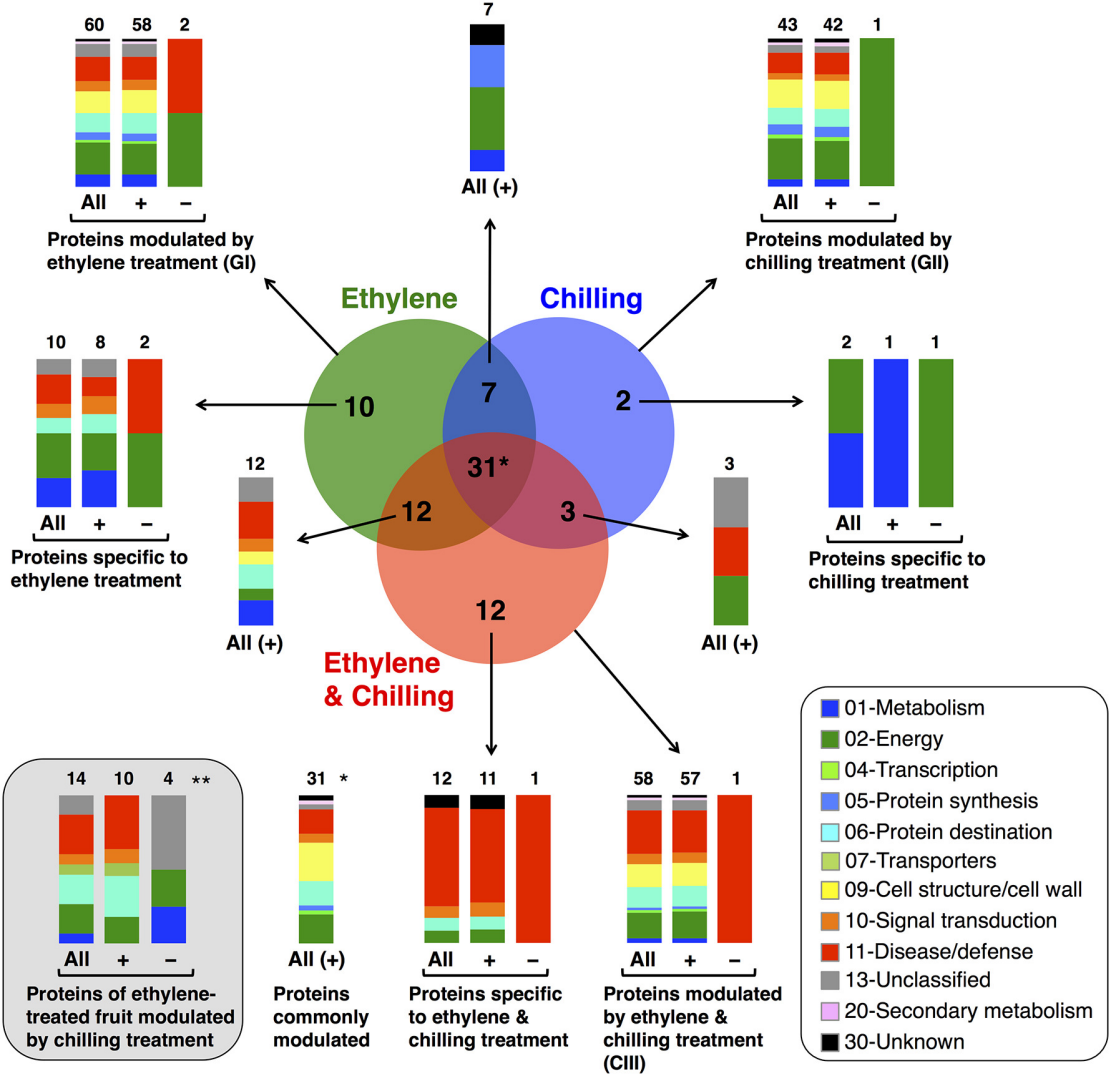
**Venn diagram showing the unique and common differentially expressed proteins in kiwifruit exposed to ethylene and chilling treatments**. Venn diagram presents the differentially expressed proteins (77 proteins) after comparing the different postharvest treatments (ethylene, chilling and ethylene and chilling) with the control fruit. The total number in each unique or overlapping set of proteins is shown. In each case, the functional classification and the relative proportion of the nanoLC-MS/MS-identified proteins in kiwifruit exposed to postharvest treatments are presented. (^*^) Indicates the number of proteins that were commonly modulated by all treatments compare to control fruit. (^**^) Indicates the identified proteins of ethylene-treated fruit that were moduladed by the chilling treatment and this comparison revealed 14 proteins the abundance of which was statistically changed. Ten out of the fourteen proteins were included to the previous comparisons (to the Venn diagram) whereas four of them were exclusively appear at this comparison. Hence, in total in this study following these two comparison approaches we were able to identify 81 kiwifruit proteins (77+4 = 81) that modulated by treatments. (+) and (−) symbols indicate identified kiwifruit proteins that were increased or decreased in abundance, respectively.

To identify proteins that accumulated differentially in kiwifruit exposed to postharvest treatments compared with control, three general trends in the data were documented, as displayed in Figure 3. The first group is represented by 60 proteins the abundance of which was changed by ethylene while the second group contains 43 proteins the abundance of which was changed by chilling treatment. Group III consists of 58 proteins that were modulated following combined ethylene and chilling treatment (Figure 3; Table 1). Functional analysis disclosed that ethylene-responsive proteins are mainly associated with energy (21.7%) followed by disease/defense (16.7%) and protein destination/storage (13.3%). Chilling-responsive proteins are mainly involved in energy (27.9%), cell structure/cell wall (18.6%) and disease/defense (14.0%). Meanwhile, the ethylene and chilling-responsive proteins are predominantly participating in disease/defense (29.3%), energy (17.2%), cell structure/cell wall (15.5%) and protein destination/storage (13.8%).

Remarkably, from the total 81 identified proteins that changed following the applied treatments (ethylene, chilling, and ethylene and chilling) compared with control, 31 proteins were commonly targeted by the three postharvest treatments, indicating the significant impact of both ethylene and chilling in kiwifruit ripening. The commonly targeted proteins were mainly involved in cell structure/cell wall (25.8%), energy (19.4%), protein destination/storage (16.1%) and disease/defense (16.1%; Figure 3). The Venn diagram also showed that among the ethylene-responsive proteins, 10 proteins were exclusively identified in ethylene-treated fruit, 7 and 12 proteins were also identified in chilling and ethylene and chilling treatments, respectively. Amongst the 42 chilling-responsive proteins, two proteins were specifically detected in chilling-treated kiwifruit, while seven and three proteins were also identified either in response to ethylene or ethylene and chilling treatments, respectively. Additionally, 12 proteins were exclusively targeted by the combined ethylene and chilling treatment, whereas 12 and 3 ethylene and chilling-responsive proteins overlapped with identified proteins in the single ethylene and chilling treatments, respectively.

Proteins associated with energy (30%), metabolism (20%) and disease/defense (20%) were predominant in the group of proteins that were identified only in ethylene treatment (*n* = 10; Figure 3). Proteins that were specifically targeted by chilling (*n* = 2) were involved in metabolism and energy. Interestingly, 9 (75%) out of 12 proteins that exclusively identified in fruit exposed to ethylene and chilling treatment were involved in disease/defense. Proteins that were commonly targeted by ethylene and ethylene and chilling treatment (*n* = 12) were mainly involved in disease/defense (25.0%), protein destination/storage (16.7%) and metabolism (16.7%). On the other hand, the overlap between ethylene and chilling treatments included seven proteins that participate mainly in energy (42.8%) and protein synthesis (28.5%), while the overlap between chilling and ethylene and chilling treatments included three proteins that associated with energy (enolase) and disease/defense (kiwellin). Additionally, as an attempt to distinguish kiwifruit proteins modulated by chilling in the group of ethylene-treated fruit, comparisons among ethylene-treated fruit ripened under non-chilling conditions and ethylene-treated fruit ripened following chilling exposure revealed 14 proteins the abundance of which changed; these proteins were mainly involved in disease/defense (26.7%), energy (20.0%), and protein destination/storage (20.0%; Figure 3).

**Figure 4:**
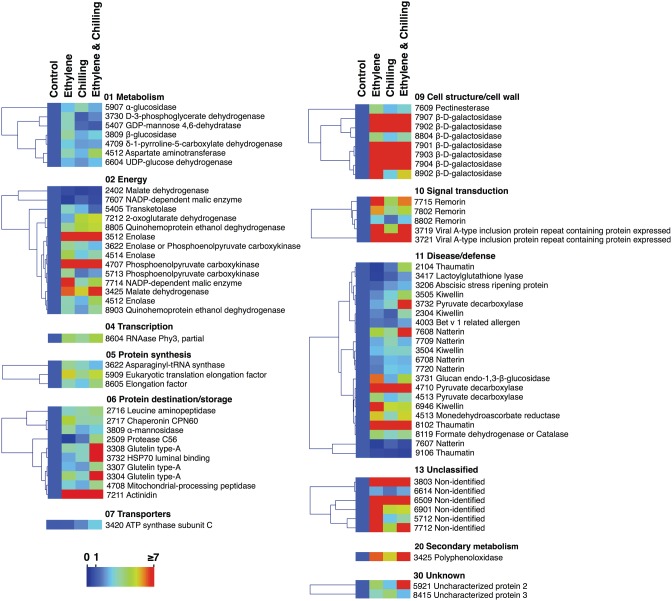
**Protein abundance changes in kiwifruit exposed to ethylene and chilling treatments**. The graph shows the relative level of each protein compared to the abundance of control. Fold change values are shown on a color scale (shown at the bottom), which is proportional to the abundance of each identified protein. Mean values of three independent determinations for each treatment were expressed as ratios between the treatment and control using the Multi-Experiment Viewer software (version 4.4.1). Relative values for each protein abundance is provided in Supplementary Table S1. Proteins were grouped according to their known functional role as given in Figure 2.

It is notable that out of the 77 proteins changed by the three treatments compared with control the main proportion of them (73) were increased in abundance, while only four were down-regulated (Figures 3, 4). All the commonly affected proteins by the three treatments were up-regulated, while down-regulated proteins detected among the ethylene-specifically affected proteins (*n* = 2, energy and disease/defense), chilling (*n* = 1, energy) and the combined ethylene and chilling treatment (*n* = 1, disease/defense). On the other hand, of the 15 identified kiwifruit proteins modulated by chilling in the ethylene-treated fruit, 11 proteins were up-regulated in abundance, while four down-regulated and these proteins were mainly involved in energy and metabolism.

### Regulatory networks of ethylene- and chilling-responsive kiwifruit proteins

**Figure 5:**
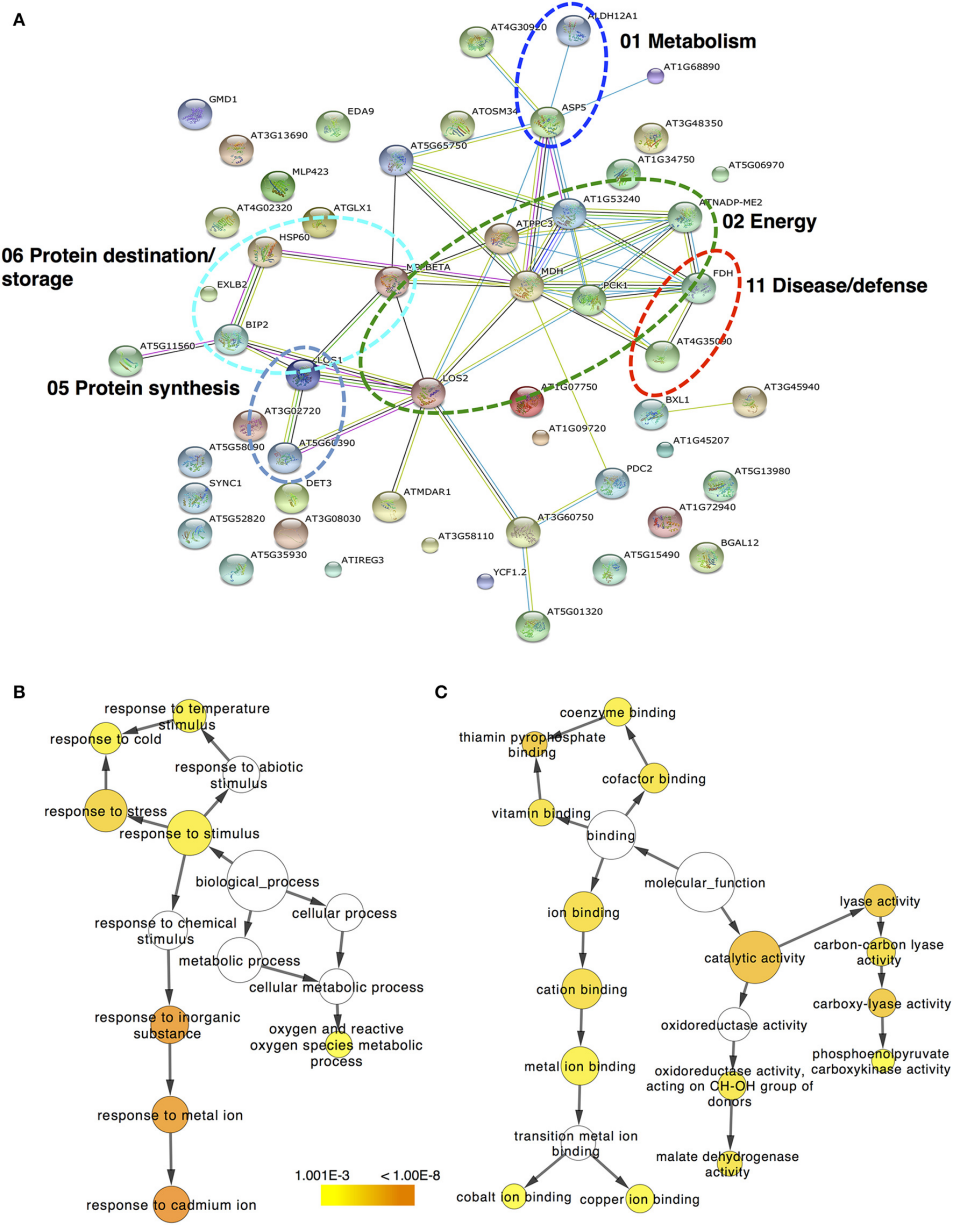
**The protein–protein interaction network simulated by STRING. (A)** Protein–protein interaction is presented for the identified proteins in kiwifruit undergo ripening. *Arabidopsis thaliana* and confidence level of 0.4 were used for analysis parameters. Different line colors represent the types of evidence used in predicting the associations: gene fusion (red), neighborhood (green), co-occurrence across genomes (blue), co-expression (black), experimental (purple), association in curated databases (light blue), or co-mentioned in PubMed abstracts (yellow). Six clusters of highly interacting protein nodes are marked with oval dotted lines and include proteins involved in metabolism, energy, protein synthesis, protein destination, and disease/defense functional categories. Biological pathway **(B)** and molecular function **(C)** networks generated by BiNGO. GO categories of TAIR homologous proteins are presented in kiwifruit undergo ripening. The size of the node is related to the number of proteins and the color represents the *p*-value for the statistical significance of the overrepresented GO term.

The protein–protein interaction network generated with STRING 9.0 (Szklarczyk et al., 2011) revealed functional links between different proteins identified in kiwifruit that were exposed to ripening elicitors. In ripe kiwifruit, the major clusters of interacting proteins are highlighted with circles in Figure 5A and involve proteins that are associated with energy, protein destination/storage, protein synthesis, metabolism and disease/defense. To obtain statistically over- or under-represented categories of predicted biological pathways and molecular functions related to kiwifruit ripening, the BiNGO 2.44 (Maere et al., 2005) software was used (Figures 5B,C). A complete list of the enriched Gene Ontology (GO) biological pathway and molecular function of identified proteins is presented in Supplementary Tables S4, S5, respectively. The most significantly over-represented biological pathways in ripe kiwifruit was the response to inorganic substance (*p* = 6.67E-08) and the response to cadmium and metal ion (*p* = 3.16E-08 and 9.52E-08, respectively). Three other major groups that should be highlighted in the present analysis are the response to stress (1.23E-05) and the response to cold (2.48E-04) along with oxygen and reactive oxygen species metabolic process (7.32E-04; Figure 5B; Supplementary Table S4). The most highly enriched molecular functions of ripening kiwifruit was the catalytic activity (*p* = 2.04E-06), carboxy-lyase activity, lyase activity and thiamin pyrophosphate binding (*p* = 4.95E-06; Figure 5C; Supplementary Table S5).

## Discussion

### Ethylene and chilling elicited climacteric kiwifruit ripening

Physiological data suggests that exogenous ethylene considerably induced kiwifruit ripening, as documented by the climacteric increase in RR, firmness and TA reduction as well as by the increase in SSC (Figure 1), thus confirming previous kiwifruit ripening studies (Antunes and Sfakiotakis, 2002; Mworia et al., 2012). In addition, exogenous ethylene provoked the autocatalytic ethylene production that was accompanied by increased ACS and ACO activities, which concomitantly increased ACC and MACC steady-levels (Figure 1). Meanwhile, chilling, in the absence of exogenous ethylene, was able to induce a burst in ethylene production and the ripening features of kiwifruit (Figure 1), indicating that chilling also elicited kiwifruit ripening independently to ethylene. The current data also showed that the impact of combined ethylene and chilling treatment in kiwifruit ripening was stronger than the individual ethylene or chilling application, as evidenced by the patterns of pericarp and core tissue firmness (Figure 1) as well as by the ethylene output and its biosynthetic pathway following 10 days at 20°C (Figure 1). It is noted that respiration rate was induced by chilling treatment (0 days at 20°C following a 10 days exposure at 0°C; Figure 1), denoting differences on kiwifruit ripening regulation mechanism under chilling and exogenous ethylene conditions. Overall, physiological data of kiwifruit ripening behavior provide an interesting experimental system to study the ripening mechanisms regulated by ethylene and/or chilling.

### Evidence for a link between ethylene and chilling signaling during kiwifruit ripening

The identification of proteins that are commonly or differentially regulated by ethylene and/or chilling is a crucial step to elucidate the mechanisms underlying kiwifruit ripening. The present proteomic analysis allowed the identification of 81 kiwifruit proteins that changed in abundance following the treatments applied (Figure 2; Table 1). It is noteworthy that the majority of these proteins (73 proteins) were up-regulated (Figure 2; Table 1), suggesting that the increase of protein pools is essential for kiwifruit ripening. In addition, the current analysis indicated that 60 or 43 proteins were changed in abundance in response to ethylene or chilling, respectively, while the abundance of 58 proteins was altered following the combination of these treatments (ethylene and chilling; Figure 3; Table 1). In addition, we found a considerable overlap between the two treatments since 31 of the total 77 ethylene/chilling-responsive proteins were commonly regulated (Figure 3; Table 1), indicating a link between ethylene and chilling signaling during kiwifruit ripening. As a result of the positive interaction between ethylene and chilling, activation of ethylene responses should render kiwifruit more sensitive to chilling-dependent ripening and *vice versa*. It is also interesting to note that approximately 74% (*n* = 60) of all identified kiwifruit proteins changed their abundance in response to exogenous ethylene while nearly 97% (*n* = 58) of these ethylene-responsive proteins were up-regulated (Figure 3; Table 1), thus providing an explanation why the ripening status of kiwifruit was remarkably affected by exogenous ethylene.

### Kiwifruit proteins exclusively affected by exogenous ethylene

Kiwifruit metabolism is different from other fruit species as carbon is mainly stored as starch and it is converted almost entirely to CO_2_ and/or sugars when fruit reaches maturity (Nardozza et al., 2013). Thus, a reasonably accurate carbon balance can be developed for kiwifruit ripening in which respiration represents glycolytic carbon flux and the rate of sugar accumulation represents gluconeogenic carbon flux. In the presented study, both the rate of CO_2_ production through respiration and the abundance of several gluconeogenesis-related proteins, including phosphoenolpyruvate carboxykinase, malate dehydrogenase, NADP-dependent malic enzyme and pyruvate decarboxylase were elevated following ethylene treatment (Figure 4; Table 1), suggesting that carbon is simultaneously being shunted in both gluconeogenic (toward sucrose synthesis) and glycolytic (toward CO_2_ synthesis) directions in kiwifruit experiencing exogenous ethylene conditions.

Mitochondrial-processing peptidase (MPP), which is required for the maturation of imported nuclear encoded mitochondrial protein precursors, was specifically induced by exogenous ethylene (Figure 4; Table 1). The proper mitochondrial function requires not only the precise and timed production and targeting of hundreds of proteins but also their correct sub-mitochondrial location, proper folding and often the correct assembly into multimeric complexes (Becker et al., 2012). Even though the relation between MPP and climacteric ripening has not been previously addressed, it is likely that the induction of MPP is essential to ensure the correct maturation of mitochondrial proteins during ethylene-induced ripening. On the other hand, studies on cell wall metabolism in fruits have mainly focused on fruit softening, and there is a gap in the knowledge regarding the cell wall synthesis, such as the role of UDP-glucose dehydrogenase (UGD) which is involved in cell wall biosynthesis (Sato et al., 2013). In this work, the relative abundance of UGD was stimulated by ethylene (Figure 4; Table 1), suggesting that the UDP-glucuronic acid synthesis could occur at the climacteric stage. Furthermore, the up-regulation of D-3-phosphoglycerate dehydrogenase (D-3-PGDH), which is involved in oxidative phosphorylation during glycolysis, by external ethylene (Figure 4; Table 1), could be linked with climacteric ethylene signaling, because it was observed that D-3-PGDH was modulated by 1-methylcyclopropene (1-MCP), an inhibitor of ethylene perception, in ripe peach fruit (Zhang et al., 2012).

### Kiwifruit proteins exclusively affected by chilling treatment

Several kiwifruit proteins showed differential abundance levels following chilling. In particular, the fact that α-glucosidase, which participates in abscisic acid (ABA) synthesis via hydrolysis of ABA-glucose ester, was induced by chilling (Figure 4; Table 1), was consistent with data showing that exogenous ABA treatment promoted ethylene production in “Hayward” fruit (Kunsong et al., 1997), possibly indicating that chilling could regulate kiwifruit ripening response by controlling ABA levels. In contrast to the results obtained above for the gluconeogenic pathway in ethylene-treated fruit, the present result indicated that chilling might postpone gluconeogenesis by inhibiting malate dehydrogenase (Figure 4; Table 1), an enzyme of the citric acid cycle that catalyzes the conversion of malate into oxaloacetate which is also involved in gluconeogenesis. Hence, the role of gluconeogenesis in kiwifruit ripening is specific, possibly reflecting opposite operations between ethylene and chilling signaling.

### Kiwifruit proteins affected by either individual or combined ethylene and chilling treatments

In addition to the above results showing distinct regulation of protein abudance by ethylene and chilling, we found a set of seven proteins which were commonly regulated in response to individual ethylene and chilling application (Figure 3; Table 1). Predominantly, we have observed a widespread increase in abudance of this group of proteins, including aspartate transaminase, asparaginyl-tRNA synthase, uncharacterized protein 3 and elongation factor 1a (Figure 3; Table 1). Aspartate transaminase catalyzes the reversible transfer of a α-amino group between aspartate and glutamate whereas asparaginyl-tRNA synthase belongs to the family of ligases and participates in aspartate metabolism. Thus, the coordinated induction of these proteins either by chilling or ethylene could regulate aspartate and glutamate homeostasis, both implicated in umami taste during the climacteric ripening transition (Sorrequieta et al., 2013).

This study also identified 12 kiwifruit proteins that were affected by ethylene and chilling treatment with 11 proteins of which being up-regulated, including Bet v 1 related allergen, natterin and abscisic stress ripening protein (Figures 3, 4; Table 1). The observed increase in abudance of bet v 1 related allergen provides evidence for a role for Bet v 1 in kiwifruit ripening as a carrier of various intermediates generated by the ripening process into different target compartments, as previously suggested in strawberry fruit (Hjernø et al., 2006). Natterins are a recently discovered gene family from the venom gland of the fish *Thalassophryne nattereri*; however, it has also been isolated from *Salvelinus alpinus* fish (Steinhäuser, 2013). Although the function of this protein has not been described in higher plants, future research is needed to characterize the function of natterins during fruit ripening.

### Kiwifruit proteins commonly affected by the ripening inductive ethylene or/and chilling treatments

The current proteomic analysis revealed a strong overlap in proteins regulated by the three treatments since 31 kiwifruit proteins were commonly sensitive to all (Figure 3; Table 1). The fact that the majority of these proteins increased in abundance might be interpreted as a general stimulation of the metabolic activity of kiwifruit during ripening that was elicited by ethylene and chilling. For instance, the induction of enolase and phosphoenolpyruvate carboxykinase (both related to glycolysis), quinohemoprotein ethanol dehydrogenase (related to electron transport chain) as well as 2-oxoglutarate dehydrogenase (related to TCA cycle) by all postharvest treatments suggests that major carbohydrate energy-releasing pathways are activated in kiwifruit undergoing ripening. In addition, fruit ripening has also been described as an endogenous oxidative process whereby ROS (reactive oxygen species) accumulation is balanced by the activity of cellular antioxidant systems. In this regard, the induction of monodehydroascorbate reductase, an enzymatic component of the ascorbate–glutathione cycle, implies that ethylene and chilling could activate this cycle in order to reduce ROS generation caused by ripening.

Kiwifruit ripening is characterized by a fast solubilisation of both pectic and cross-linking glycans coupled with an increase in the viscosity of the cell wall material (Minas et al., 2014). The enzyme β-galactosidase (β-gal) was suggested to be involved in cell wall swelling and softening in kiwifruit (Gallego and Zarra, 1997); however, this β-gal action was not fully supported by other studies (Redgwell et al., 1992). The fact that the seven isoforms of β-gal were induced by all treatments (Figure 2C plate 2, Figure 4; Table 1) suggested that kiwifruit softening is highly dependent on β-gal activation. Another induced protein in kiwifruit exposed to ripening elicitors was the “Viral A-type inclusion protein repeat containing protein expressed” (Figure 4; Table 1). Viral A-like proteins represent a collection of poorly characterized eukaryotic proteins mostly identified from similarity to pox virus proteins that generate inclusion bodies in the host cytoplasm into which viral A-like particles are sequestered (Gould et al., 2011). Almost nothing is known about their function in eukaryotes, but one viral A-like protein of the protist *Trichomonas* sp. (p477, TVAG_012450) has been implicated in cytoskeleton remodeling (Bricheux et al., 2007) which may be correlated with the differences in kiwifruit softening patterns (Figure 1). Remorins are plant-specific proteins present in all land plants and which are exclusively detected in detergent-insoluble membrane fractions (commonly called lipid rafts). Several members of the remorin family were found to be regulated during plant-microbe interactions (Widjaja et al., 2009); however, no roles had been assigned to remorin proteins during ripening until now. Thus, the up-regulation of three remorin isoforms during kiwifruit ripening by all postharvest treatments (Figure 2C plate 1, Figure 5; Table 1) suggests that remorin plays an important role in climacteric ripening process. Future research will be focused on the characterization of the specific function of remorins in fruit ripening.

### Interaction network of differentially accumulated proteins in ripen kiwifruit

Proteins in a living cell do not act as single entities, but they form a variety of functional connections with each other which are fundamental in cellular processes (Miernyk and Thelen, 2008). Bioinfomatic approach using STRING 9.0 (Szklarczyk et al., 2011) allowed the characterization of the main cluster of energy-related proteins, such as enolase, which interacts with four other clusters of metabolism-related proteins (e.g., aspartate aminotransferase), disease/defense-related (e.g., catalase), protein destination/storage-related proteins (e.g., HSP70 luminal binding) and protein synthesis-related proteins (e.g., elongation factor; Figure 5A; Supplementary Table S3). Enolase (LOS2 in Figure 5A; Supplementary Table S3), which catalyzes the conversion of 2-phosphoglycerate to phosphoenolpyruvate, was the central core protein in this interacting network. Thus, the strong induction of enolase by both ethylene and chilling (Figure 4; Table 1) could be associated with climacteric fruit ripening, further supported by the enolase accumulation in tomato fruit following ethylene treatment (Zegzouti et al., 1999). In addition, bioinformatic analysis using BiNGO (Maere et al., 2005) predicted the main molecular functions of proteins identified in ripe kiwifruit, including catalytic activity (34 proteins), carboxy-lyase activity (five proteins), lyase activity (eight proteins) and thiamin pyrophosphate binding (three proteins; Figure 5C; Supplementary Table S5). The bioinfomatic approach also indicated that the response to inorganic substance/metal ion was the most considerably affected biological pathway (Figure 5B; Supplementary Table S4), indicating that these metabolic processes play important roles in kiwifruit ripening. This finding is consistent with previously bioinformatic analysis showing that the responses to inorganic compounds and metal ion cofactors are significant biological processes in pineapple fruit ripening (Koia et al., 2012). Being incorporated into or associated with proteins (Tan et al., 2010), inorganic substance/metal ion could be important in fruit ripening because they elicit various ripening-related functions, including electron transport, ATP synthesis and ROS detoxification (Molassiotis et al., 2013). Strong evidence also suggest that the mechanism of ethylene production involves metal chelation while (Abeles et al., 1992) ethylene signaling is mediated by a family of high-affinity metal-containing receptors through a pathway that includes metallic transporter intermediates leading to downstream ripening signaling (Stepanova and Alonso, 2005). Proteins classified in these biological pathways following the general category of response to chemical stimulus consisted of several proteins, including lactoylglutathione lyase, enolase, transketolase, aspartate aminotransferase, monodehydroascorbate reductase, chaperonin CPN60 etc. Three other biological pathways over-represented in kiwifruit included the response to stress (16 proteins), to temperature stimulus (seven proteins) and to cold (six proteins; Figure 5B; Supplementary Table S4), consistent with the experimental procedure followed in the present study (Supplementary Figure S1).

In summary, we have found that exogenous ethylene or chilling treatments elicited kiwifruit ripening. This study further provides insight into the ethylene- and chilling-originated ripening in terms of protein change signatures. Protein targets of ethylene or/and chilling are involved in a wide range of metabolic pathways, such as disease/defense, energy, protein destination/storage and cell structure/cell wall, also suggesting the impact of these ripening elicitors to various previously unknown proteins. In particular, ethylene and chilling appear to regulate MPP, natterins, a Bet v 1 related allergen, Viral A-type inclusion protein repeat containing protein expressed, enolase and remorins. Focused studies on specific enzymes as well as systems-wide transcriptomic and metabolomic studies are needed to further decipher the ethylene- and chilling-associated kiwifruit ripening. This approach will also enable the future large scale analysis at pre-climacteric stage to explain early differences and common links of kiwifruit ripening in response to two stimuli.

## Author contributions

IM and AM designed the study. IM, GT, EK, and MB carried out the experimental work and data analysis. IM performed graph artwork, wrote and prepared the first draft of the manuscript. AM substantially improved the first draft of the manuscript. GT and MB edited the other versions. All the authors have read and approved this manuscript.

### Conflict of interest statement

The authors declare that the research was conducted in the absence of any commercial or financial relationships that could be construed as a potential conflict of interest.

## Supplementary material

Supplementary Table S1 **Quantitative analysis of protein spot volumes**.

Supplementary Table S2 **Sequence data of kiwifruit proteins analyzed by 2DE-PAGE and nanoLC-MS/MS**.

Supplementary Table S3 **Identified kiwifruit proteins Blasted against the TAIR database and their STRING 9.0 ID**.

Supplementary Table S4 **Biological pathways generated by BINGO in kiwifruit**.

Supplementary Table S5 **Molecular functions generated by BINGO in kiwifruit**.

Supplementary Figure S1 **Experimental design**.
