# Supplementary material for: Coupling of Physiological and Proteomic Analysis to Understand the Ethylene- and Chilling-Induced Kiwifruit Ripening Syndrome
Source: Front Plant Sci. 2016 Feb 15;7:120. doi: 10.3389/fpls.2016.00120 (PMC4753329; doi:10.3389/fpls.2016.00120)
Supplement: Supplementary Table S1 — Quantitative analysis of protein spot volumes. [file Table1.docx]

| **Supplementary Table S1** | | | | | | | | |
| --- | --- | --- | --- | --- | --- | --- | --- | --- |
| **Sequence data of kiwifruit proteins identified in the flesh tissue.  Proteins have been analyzed by two-dimensional electrophoresis and identified by mass spectrometry LC/MS-MS.  Legend:  Spot No, spot label on the reference gel maps presented in Figure 2;  Protein name, identified peptide names;  Acces. number, accession number in NCBI or Kiwifruit Genome database; Matching criteria, adopted criteria for the valuation of similarity for proteins identified after manual blast against current databases. Blank cells correspond to proteins readily identified without further blast;  Functional category, proteins ontologically classified into functional categories proposed by Bevan et al. (1998). Nature, 391, 485-488; % Cov, Mascot coverage %, percentage of sequence coverage obtained with identified peptides with Mascot software for the orthologous protein; Score, a minimum of two different peptides was used (score>52 for search in NCBI nr, score>31 for search in KIWIFRUIT GENOME and score>49 when using the EST database); When presented identifications based on single peptide additional information are provided. m/z; Mass (exp); Sequences, list of peptides identified as mentioned in Material and Methods.** | | | | | | | | |
| **Spot Νο** | **Protein name** | **Accession number in NCBI or Kiwifruit Genome database** | **Matching criteria** | **% cov** | **Score** | **m/z** | **Mass (exp)** | **Sequences** |
| 2104 | Thaumatin | gi\|190358875 |  | 16 | 1134 | 527,7210 | 1053,4273 | R.TGCNFDGAGR.G + Carbamidomethyl (C) |
|  |  |  |  |  |  | 527,7218 | 1053,4290 | R.TGCNFDGAGR.G + Carbamidomethyl (C) |
|  |  |  |  |  |  | 527,7219 | 1053,4292 | R.TGCNFDGAGR.G + Carbamidomethyl (C) |
|  |  |  |  |  |  | 527,7219 | 1053,4292 | R.TGCNFDGAGR.G + Carbamidomethyl (C) |
|  |  |  |  |  |  | 527,7220 | 1053,4295 | R.TGCNFDGAGR.G + Carbamidomethyl (C) |
|  |  |  |  |  |  | 527,7221 | 1053,4297 | R.TGCNFDGAGR.G + Carbamidomethyl (C) |
|  |  |  |  |  |  | 527,7221 | 1053,4297 | R.TGCNFDGAGR.G + Carbamidomethyl (C) |
|  |  |  |  |  |  | 527,7221 | 1053,4297 | R.TGCNFDGAGR.G + Carbamidomethyl (C) |
|  |  |  |  |  |  | 527,7221 | 1053,4297 | R.TGCNFDGAGR.G + Carbamidomethyl (C) |
|  |  |  |  |  |  | 527,7221 | 1053,4297 | R.TGCNFDGAGR.G + Carbamidomethyl (C) |
|  |  |  |  |  |  | 527,7222 | 1053,4299 | R.TGCNFDGAGR.G + Carbamidomethyl (C) |
|  |  |  |  |  |  | 527,7222 | 1053,4299 | R.TGCNFDGAGR.G + Carbamidomethyl (C) |
|  |  |  |  |  |  | 527,7223 | 1053,4300 | R.TGCNFDGAGR.G + Carbamidomethyl (C) |
|  |  |  |  |  |  | 527,7224 | 1053,4303 | R.TGCNFDGAGR.G + Carbamidomethyl (C) |
|  |  |  |  |  |  | 527,7224 | 1053,4303 | R.TGCNFDGAGR.G + Carbamidomethyl (C) |
|  |  |  |  |  |  | 527,7227 | 1053,4308 | R.TGCNFDGAGR.G + Carbamidomethyl (C) |
|  |  |  |  |  |  | 527,7230 | 1053,4314 | R.TGCNFDGAGR.G + Carbamidomethyl (C) |
|  |  |  |  |  |  | 527,7230 | 1053,4315 | R.TGCNFDGAGR.G + Carbamidomethyl (C) |
|  |  |  |  |  |  | 527,7231 | 1053,4316 | R.TGCNFDGAGR.G + Carbamidomethyl (C) |
|  |  |  |  |  |  | 678,3578 | 1354,7010 | R.GQNWIINPGAGTK.G |
|  |  |  |  |  |  | 678,3581 | 1354,7016 | R.GQNWIINPGAGTK.G |
|  |  |  |  |  |  | 711,3195 | 1420,6244 | R.APGGCNNPCTVFK.T + 2 Carbamidomethyl (C) |
| 2304 | Kiwellin | gi\|441482352 |  | 40 | 4256 | 490,2111 | 978,4077 | K.VVDECDSR.H |
|  |  |  |  |  |  | 726,3437 | 1450,6728 | K.SVSAKVVDECDSR.H |
|  |  |  |  |  |  | 524,8890 | 1571,6453 | K.CNDDPEVGTHICR.G |
|  |  |  |  |  |  | 786,8309 | 1571,6472 | K.CNDDPEVGTHICR.G |
|  |  |  |  |  |  | 875,3937 | 1748,7729 | R.DLNDCDGQLICIEGK.C |
|  |  |  |  |  |  | 644,6314 | 1930,8724 | K.SHPTYDCSPPVTSSTPAK.L |
|  |  |  |  |  |  | 966,4442 | 1930,8737 | K.SHPTYDCSPPVTSSTPAK.L |
|  |  |  |  |  |  | 1015,3937 | 3043,1594 | K.LTNNDFSEGGDGGGPSECDESYHSNNER.I |
| 2402 | Malate dehydrogenase | Achn121571 |  | 9 | 318 | 339,6765 | 677,3385 | K.GVYDPK.K |
|  |  |  |  |  |  | 419,2271 | 836,4396 | R.FVESSLR.A |
|  |  |  |  |  |  | 440,2322 | 878,4499 | R.ANTFVAEK.K |
|  |  |  |  |  |  | 481,2531 | 960,4917 | R.LQPPESYK.V |
|  |  |  |  |  |  | 629,8356 | 1257,6566 | R.IQNAGTEVVEAK.A |
| 2509 | Protease C56 | Achn264591 |  | 7 | 81 | 323,1999 | 644,3852 | K.ALGGTVK.G |
|  |  |  |  |  |  | 323,2003 | 644,3860 | K.ALGGTVK.G |
|  |  |  |  |  |  | 323,2005 | 644,3864 | K.ALGGTVK.G |
|  |  |  |  |  |  | 346,1633 | 690,3121 | K.AGDICR.T + Carbamidomethyl (C) |
|  |  |  |  |  |  | 346,1634 | 690,3122 | K.AGDICR.T + Carbamidomethyl (C) |
|  |  |  |  |  |  | 346,1634 | 690,3122 | K.AGDICR.T + Carbamidomethyl (C) |
|  |  |  |  |  |  | 455,2285 | 908,4423 | K.CTAYPAVK.L + Carbamidomethyl (C) |
|  |  |  |  |  |  | 455,2286 | 908,4427 | K.CTAYPAVK.L + Carbamidomethyl (C) |
|  |  |  |  |  |  | 475,7564 | 949,4982 | R.TAIQQFSR.H |
| 2711 | Chaperonin CPN60 | gi\|356534856 |  | 11 | 293 | 515,7976 | 1029,5806 | K.GVEELADAVK.V |
|  |  |  |  |  |  | 573,8141 | 1145,6136 | K.IGGASEAEVGEK.K |
|  |  |  |  |  |  | 618,3290 | 1234,6434 | K.SVAAGMNAMDLR.R |
|  |  |  |  |  |  | 644,8765 | 1287,7384 | R.NVVIEQSFGAPK.V |
|  |  |  |  |  |  | 644,8807 | 1287,7468 | R.NVVIEQSFGAPK.V |
|  |  |  |  |  |  | 1118,0906 | 2234,1666 | K.QVANATNDVAGDGTTCATVLTR.A + Carbamidomethyl (C) |
| 2716 | Leucine aminopeptidase | Achn369241 |  | 21 | 337 | 636,3386 | 1270,6626 | K.GLTFDSGGYNIK.T |
|  |  |  |  |  |  | 647,8562 | 1293,6978 | K.FDMGGSAAVLGAAK.A |
|  |  |  |  |  |  | 727,8937 | 1453,7728 | K.YTEDVCSGIILGK.E + Carbamidomethyl (C) |
|  |  |  |  |  |  | 759,4399 | 1516,8652 | K.SVDILGLGTGSELEK.K |
|  |  |  |  |  |  | 855,9942 | 1709,9738 | R.VGLIGLGQSSSTTTAYR.S |
|  |  |  |  |  |  | 855,9975 | 1709,9804 | R.VGLIGLGQSSSTTTAYR.S |
| 3206 | Abscisic stress ripening protein | Achn305291 |  | 12 | 429 | 487,2166 | 972,4187 | K.TGEFGDYGK.S |
|  |  |  |  |  |  | 624,2622 | 1246,5099 | K.TDGYGGGYGGESK.T |
| 3304 | Glutelin type-A | gi\|195225381 | 57% to gi\|225447983 Vitis vinifera, E value:1e-70 | 21 | 196 | 643,8571 | 1285,6996 | K.GTPGNENELLDK.Q |
|  |  |  |  |  |  | 672,8760 | 1343,7374 | K.LEEGISIPQACK.G + Carbamidomethyl (C) |
|  |  |  |  |  |  | 832,4636 | 1662,9126 | K.ANEVSSPIYVADSGVR.V |
| 3307 | Glutelin type-A | Achn231381 |  | 15 | 218 | 361,6953 | 721,3760 | R.AYNLNK.E |
|  |  |  |  |  |  | 361,6953 | 721,3761 | R.AYNLNK.E |
|  |  |  |  |  |  | 361,6953 | 721,3761 | R.AYNLNK.E |
|  |  |  |  |  |  | 387,2237 | 772,4329 | R.VLDAEVK.A |
|  |  |  |  |  |  | 387,2238 | 772,4330 | R.VLDAEVK.A |
|  |  |  |  |  |  | 387,2238 | 772,4331 | R.VLDAEVK.A |
|  |  |  |  |  |  | 387,2238 | 772,4331 | R.VLDAEVK.A |
|  |  |  |  |  |  | 387,2239 | 772,4331 | R.VLDAEVK.A |
|  |  |  |  |  |  | 387,2239 | 772,4332 | R.VLDAEVK.A |
|  |  |  |  |  |  | 387,2239 | 772,4333 | R.VLDAEVK.A |
|  |  |  |  |  |  | 487,2770 | 972,5393 | K.AGDLFVVPR.F |
|  |  |  |  |  |  | 508,3105 | 1014,6065 | K.QVGLSATLVK.L |
|  |  |  |  |  |  | 508,3106 | 1014,6067 | K.QVGLSATLVK.L |
|  |  |  |  |  |  | 508,3106 | 1014,6067 | K.QVGLSATLVK.L |
|  |  |  |  |  |  | 542,8095 | 1083,6045 | R.VQIVGINGER.V |
|  |  |  |  |  |  | 643,8137 | 1285,6129 | K.GTPGNENELLDK.Q |
|  |  |  |  |  |  | 643,8138 | 1285,6131 | K.GTPGNENELLDK.Q |
|  |  |  |  |  |  | 643,8153 | 1285,6161 | K.GTPGNENELLDK.Q |
| 3308 | Glutelin type-A | gi\|195248852 | 58% to gi\|225447983 Vitis vinifera, E value:2e-82 | 51 | 542 | 307,7155 | 613,4165 | K.GALIIK.L |
|  |  |  |  |  |  | 354,7183 | 707,4220 | R.VTYVVK.G |
|  |  |  |  |  |  | 361,6952 | 721,3759 | R.AYNLNK.E |
|  |  |  |  |  |  | 387,2238 | 772,4329 | R.VLDAEVK.A |
|  |  |  |  |  |  | 408,7632 | 815,5118 | K.EKVVTIK.Q |
|  |  |  |  |  |  | 487,2771 | 972,5396 | K.AGDLFVVPR.F |
|  |  |  |  |  |  | 508,3104 | 1014,6062 | K.QVGLSATLVK.L |
|  |  |  |  |  |  | 542,8091 | 1083,6037 | R.VQIVGINGER.V |
|  |  |  |  |  |  | 641,8174 | 1281,6203 | R.AYNLNKEESSK.F |
|  |  |  |  |  |  | 643,8149 | 1285,6152 | K.GTPGNENELLDK.Q |
|  |  |  |  |  |  | 672,8450 | 1343,6754 | K.LEEGISIPQACK.G |
|  |  |  |  |  |  | 832,4208 | 1662,8271 | K.ANEVSSPIYVADSGVR.V |
| 3417 | Lactoylglutathione lyase | Achn326191 |  | 3 | 63 | 646,8680 | 1291,7214 | K.TVLVDNEDFLK.E |
|  |  |  |  |  |  | 646,8744 | 1291,7342 | K.TVLVDNEDFLK.E |
| 3420 | ATP synthase subunit C | Achn017021 |  | 9 | 187 | 366,6924 | 731,3703 | K.IEDDLK.V |
|  |  |  |  |  |  | 387,7216 | 773,4286 | R.KQELEK.L |
|  |  |  |  |  |  | 394,7290 | 787,4435 | R.DLSNLVK.A |
|  |  |  |  |  |  | 409,2300 | 816,4455 | K.QSGSLAVR.D |
|  |  |  |  |  |  | 473,2591 | 944,5036 | R.SQLNTINR.K |
|  |  |  |  |  |  | 482,7458 | 963,4771 | R.VAEYNNVR.S |
| 3425 | Malate dehydrogenase | Achn221601 |  | 20 | 597 | 343,6791 | 685,3436 | K.TFYAGK.A |
|  |  |  |  |  |  | 432,2362 | 862,4579 | K.SLCTAIAK.Y |
|  |  |  |  |  |  | 432,7322 | 863,4498 | K.GIKFANQS.- |
|  |  |  |  |  |  | 456,2266 | 910,4386 | K.KAGTYDEK.K |
|  |  |  |  |  |  | 456,2267 | 910,4387 | K.AGTYDEKK.L |
|  |  |  |  |  |  | 617,3016 | 1232,5886 | R.TQDGGTEVVEAK.A |
|  |  |  |  |  |  | 659,8550 | 1317,6955 | R.DDLFNINAGIVK.S |
|  |  |  |  |  |  | 695,3518 | 1388,6889 | K.RTQDGGTEVVEAK.A |
|  |  |  |  |  |  | 752,8782 | 1503,7418 | K.ANNLSDEDITALTK.R |
|  |  |  |  |  |  | 830,9285 | 1659,8425 | K.ANNLSDEDITALTKR.T |
|  |  |  |  |  |  | 554,2883 | 1659,8430 | K.ANNLSDEDITALTKR.T |
|  | Polyphenoloxidase | Achn226891 |  | 10 | 470 | 435,7577 | 869,5008 | K.IDVPWLK.S |
|  |  |  |  |  |  | 467,2503 | 932,4861 | K.VKDSLDEK.K |
|  |  |  |  |  |  | 467,7188 | 933,4229 | K.FGYEYQK.I |
|  |  |  |  |  |  | 492,7521 | 983,4896 | K.LFMGTPYR.A |
|  |  |  |  |  |  | 500,7498 | 999,4851 | K.LFMGTPYR.A |
|  |  |  |  |  |  | 515,7979 | 1029,5811 | R.QVVSSGKTPK.L |
|  |  |  |  |  |  | 344,2011 | 1029,5813 | R.QVVSSGKTPK.L |
|  |  |  |  |  |  | 643,8062 | 1285,5977 | K.LMNELPDSDPR.S |
|  |  |  |  |  |  | 651,8033 | 1301,5920 | K.LMNELPDSDPR.S |
|  |  |  |  |  |  | 757,3680 | 1512,7214 | R.YANTNSPLYSDIR.D |
| 3504 | Kiwellin | gi\|85701136 |  | 49 | 937 | 469,6991 | 937,3836 | -.ISSCNGPCR.D |
|  |  |  |  |  |  | 490,2324 | 978,4502 | K.VVDECDSR.H + Carbamidomethyl (C) |
|  |  |  |  |  |  | 490,2349 | 978,4552 | K.VVDECDSR.H + Carbamidomethyl (C) |
|  |  |  |  |  |  | 490,7369 | 979,4592 | K.VVDECDSR.H + Carbamidomethyl (C) |
|  |  |  |  |  |  | 525,7252 | 1049,4358 | -.ISSCNGPCR.D + 2 Carbamidomethyl (C) |
|  |  |  |  |  |  | 525,7332 | 1049,4518 | -.ISSCNGPCR.D + 2 Carbamidomethyl (C) |
|  |  |  |  |  |  | 526,2478 | 1050,4810 | -.ISSCNGPCR.D + 2 Carbamidomethyl (C) |
|  |  |  |  |  |  | 582,3339 | 1162,6532 | R.ITASNGKSVSAK.V |
|  |  |  |  |  |  | 582,3465 | 1162,6784 | R.ITASNGKSVSAK.V |
|  |  |  |  |  |  | 741,3922 | 1480,7698 | R.IVALSTGWYNGGSR.C |
|  |  |  |  |  |  | 741,4053 | 1480,7960 | R.IVALSTGWYNGGSR.C |
|  |  |  |  |  |  | 741,4074 | 1480,8002 | R.IVALSTGWYNGGSR.C |
|  |  |  |  |  |  | 741,4119 | 1480,8092 | R.IVALSTGWYNGGSR.C |
|  |  |  |  |  |  | 782,3492 | 1562,6838 | R.DLNDCDGQLICIK.G + 2 Carbamidomethyl (C) |
|  |  |  |  |  |  | 782,3917 | 1562,7688 | R.DLNDCDGQLICIK.G + 2 Carbamidomethyl (C) |
|  |  |  |  |  |  | 782,3981 | 1562,7816 | R.DLNDCDGQLICIK.G + 2 Carbamidomethyl (C) |
|  |  |  |  |  |  | 937,0110 | 1872,0074 | R.NNIVDGSNAVWSALGLDK.N |
|  |  |  |  |  |  | 979,4913 | 1956,9680 | K.SYPTYDCSPPVTSSTPAK.L + Carbamidomethyl (C) |
|  |  |  |  |  |  | 979,4916 | 1956,9686 | K.SYPTYDCSPPVTSSTPAK.L + Carbamidomethyl (C) |
|  |  |  |  |  |  | 979,4943 | 1956,9740 | K.SYPTYDCSPPVTSSTPAK.L + Carbamidomethyl (C) |
|  |  |  |  |  |  | 980,4914 | 1958,9682 | K.SYPTYDCSPPVTSSTPAK.L + Carbamidomethyl (C) |
|  |  |  |  |  |  | 980,5087 | 1959,0028 | K.SYPTYDCSPPVTSSTPAK.L + Carbamidomethyl (C) |
|  |  |  |  |  |  | 715,0021 | 2141,9845 | R.GKSYPTYDCSPPVTSSTPAK.L + Carbamidomethyl (C) |
|  |  |  |  |  |  | 1072,0515 | 2142,0884 | R.GKSYPTYDCSPPVTSSTPAK.L + Carbamidomethyl (C) |
| 3505 | Kiwellin | gi\|85701136 |  | 14 | 109 | 741,4105 | 1480,8064 | R.IVALSTGWYNGGSR.C |
|  |  |  |  |  |  | 782,4051 | 1562,7956 | R.DLNDCDGQLICIK.G + 2 Carbamidomethyl (C) |
| 3512 | Enolase | gi\|14423687 |  | 9 | 175 | 641,8376 | 1281,6606 | R.DGGSDYLGKGVSK.A |
|  |  |  |  |  |  | 755,9668 | 1509,9190 | K.VQIVGDDLLVTNPK.R |
|  |  |  |  |  |  | 787,4716 | 1572,9286 | K.VNQIGSVTESIEAVK.M |
| 3622 | Enolase | Achn354501 |  | 5 | 344 | 383,1980 | 764,3814 | R.QIFDSR.G |
|  |  |  |  |  |  | 403,7296 | 805,4447 | K.YNQLLR.I |
|  |  |  |  |  |  | 456,2087 | 910,4029 | R.DGGSDYLGK.G |
|  |  |  |  |  |  | 459,7575 | 917,5005 | K.SCNALLLK.V |
|  | Asparaginyl-tRNA synthetase | Achn069631 |  | 3 | 145 | 376,7132 | 751,4119 | R.YLTEVK.F |
|  |  |  |  |  |  | 473,7692 | 945,5239 | R.TNTISAIAR.I |
|  |  |  |  |  |  | 508,2799 | 1014,5453 | K.VGELIGGSQR.E |
|  | Phosphoenolpyruvate carboxylase | Achn133831 |  | 5 | 134 | 323,6923 | 645,3700 | R.VDTALK.N |
|  |  |  |  |  |  | 401,2269 | 800,4393 | R.VTPEVTR.D |
|  |  |  |  |  |  | 408,2222 | 814,4298 | K.NIGINER.I |
|  |  |  |  |  |  | 469,6944 | 937,3742 | R.SLCACGDR.S |
|  |  |  |  |  |  | 557,8088 | 1113,6030 | K.AQEELINVAK.Q |
|  |  |  |  |  |  | 627,3464 | 1252,6783 | K.LADLDAAPAAVAR.L |
| 3719 | Viral A-type inclusion protein repeat containing protein expressed | gi\|356510118 |  | 1 | 52 | 637,3629 | 1272,7112 | K.LEEVEEELKR.V |
| 3721 | Viral A-type inclusion protein repeat containing protein expressed | gi\|356510118 |  | 1 | 60 | 637,3636 | 1272,7126 | K.LEEVEEELKR.V |
| 3730 | D-3-phosphoglycerate dehydrogenase | Achn006391 |  | 12 | 285 | 336,2320 | 670,4494 | R.LKVVGR.A |
|  |  |  |  |  |  | 343,2214 | 684,4283 | R.IINVAR.G |
|  |  |  |  |  |  | 359,1984 | 716,3822 | K.VGSEVAR.R |
|  |  |  |  |  |  | 374,7194 | 747,4243 | R.SGTKVTR.D |
|  |  |  |  |  |  | 384,2062 | 766,3979 | K.VTYASAR.A |
|  |  |  |  |  |  | 393,7456 | 785,4767 | K.LAEKLGR.L |
|  |  |  |  |  |  | 437,2478 | 872,4811 | K.VGSEVARR.A |
|  |  |  |  |  |  | 451,7142 | 901,4138 | R.APDDLDTR.L |
|  |  |  |  |  |  | 484,2274 | 966,4403 | R.DVFEASSGR.L |
|  |  |  |  |  |  | 501,7645 | 1001,5145 | R.NVAQADASVK.A |
|  |  |  |  |  |  | 525,7770 | 1049,5394 | K.ILNDETFAK.M |
| 3731 | Glucan endo-1,3-beta-glucosidase | Achn236981 |  | 8 | 285 | 393,7271 | 785,4395 | K.AGLGNQVK.V |
|  |  |  |  |  |  | 510,7636 | 1019,5127 | R.NANLAYAQR.F |
|  |  |  |  |  |  | 545,7513 | 1089,4880 | R.NNQLDQACK.F |
|  |  |  |  |  |  | 692,3196 | 1382,6247 | R.LGYGTSCGNLDAR.G |
| 3732 | Pyruvate decarboxylase | Achn036401 |  | 13 | 1090 | 348,1630 | 694,3115 | K.CWTTK.V |
|  |  |  |  |  |  | 431,7552 | 861,4958 | R.DFLGALVK.R |
|  |  |  |  |  |  | 456,2691 | 910,5237 | K.AIIVQPDR.V |
|  |  |  |  |  |  | 495,2564 | 988,4981 | K.ELLEWGSR.V |
|  |  |  |  |  |  | 509,8056 | 1017,5967 | R.DFLGALVKR.L |
|  |  |  |  |  |  | 541,8226 | 1081,6307 | K.AVKPVMVGGPK.L |
|  |  |  |  |  |  | 361,5512 | 1081,6316 | K.AVKPVMVGGPK.L |
|  |  |  |  |  |  | 366,8830 | 1097,6273 | K.AVKPVMVGGPK.L |
|  |  |  |  |  |  | 570,8242 | 1139,6338 | R.EPVPFVISPR.L |
|  |  |  |  |  |  | 384,8852 | 1151,6339 | R.IYVPEGHPLK.C |
|  |  |  |  |  |  | 409,2402 | 1224,6987 | R.VNVLFQHIQK.M |
|  |  |  |  |  |  | 613,3568 | 1224,6991 | R.VNVLFQHIQK.M |
|  |  |  |  |  |  | 466,2127 | 1395,6161 | K.QNTTAYENYHR.I |
|  |  |  |  |  |  | 698,8162 | 1395,6179 | K.QNTTAYENYHR.I |
|  | HSP70 luminal binding | Achn177881 |  | 18 | 584 | 364,7371 | 727,4596 | K.VQQLLK.E |
|  |  |  |  |  |  | 439,7586 | 877,5027 | R.TIFDVKR.L |
|  |  |  |  |  |  | 440,2205 | 878,4264 | R.SLTKDCR.L |
|  |  |  |  |  |  | 450,7792 | 899,5439 | R.NTVIPTKK.S |
|  |  |  |  |  |  | 495,2488 | 988,4831 | R.LSQEEIDR.M |
|  |  |  |  |  |  | 509,2874 | 1016,5603 | K.ITITNDKGR.L |
|  |  |  |  |  |  | 513,2778 | 1024,5411 | R.ALSSQHQVR.V |
|  |  |  |  |  |  | 528,6332 | 1582,8779 | R.QATKDAGVIAGLNVAR.I |
|  |  |  |  |  |  | 827,9482 | 1653,8818 | R.LIGEAAKNQAAVNAER.T |
|  |  |  |  |  |  | 833,4171 | 1664,8197 | K.DAVVTVPAYFNDAQR.Q |
|  |  |  |  |  |  | 596,6688 | 1786,9846 | R.IINEPTAAAIAYGLDKK.G |
|  |  |  |  |  |  | 894,5015 | 1786,9885 | R.IINEPTAAAIAYGLDKK.G |
| 3803 | Non-identified |  |  |  |  |  |  |  |
| 3809 | Beta-glucosidase | Achn262021 |  | 11 | 609 | 366,2185 | 730,4225 | R.LTLQEK.I |
|  |  |  |  |  |  | 394,2190 | 786,4234 | R.VEDLVGR.L |
|  |  |  |  |  |  | 408,2057 | 814,3969 | K.YGSAYVR.G |
|  |  |  |  |  |  | 541,7618 | 1081,5091 | R.LGFFDGDPSK.Q |
|  |  |  |  |  |  | 557,3294 | 1112,6443 | K.GSLPLSPTTIK.T |
|  |  |  |  |  |  | 586,8190 | 1171,6235 | K.AGLVNESVIDR.A |
|  |  |  |  |  |  | 649,8701 | 1297,7256 | K.TLAVIGPNADVTK.T |
|  |  |  |  |  |  | 704,3121 | 1406,6096 | K.DVCSSENQELAR.E |
|  |  |  |  |  |  | 736,3676 | 1470,7207 | R.GQETPGEDPLLTSK.Y |
|  | Alpha-mannosidase | Achn348701 |  | 4 | 462 | 387,2169 | 772,4193 | K.REDVVR.I |
|  |  |  |  |  |  | 405,1932 | 808,3719 | R.IDYQDR.A |
|  |  |  |  |  |  | 406,2212 | 810,4278 | R.QLEFFK.G |
|  |  |  |  |  |  | 495,7541 | 989,4936 | K.EQFNVTPR.I |
|  |  |  |  |  |  | 523,7485 | 1045,4825 | K.QHVADDYAK.R |
|  |  |  |  |  |  | 349,5020 | 1045,4840 | K.QHVADDYAK.R |
|  |  |  |  |  |  | 573,8115 | 1145,6085 | K.SLEVIWQGSK.S |
|  |  |  |  |  |  | 581,7816 | 1161,5487 | R.TMSAYYLASR.Q |
|  |  |  |  |  |  | 589,7792 | 1177,5438 | R.TMSAYYLASR.Q |
|  |  |  |  |  |  | 607,8169 | 1213,6192 | K.LIHYVNQDGR.V |
|  |  |  |  |  |  | 405,5477 | 1213,6214 | K.LIHYVNQDGR.V |
|  |  |  |  |  |  | 828,9286 | 1655,8426 | R.VNALYSTPTIYTDAK.Y |
| 4003 | Bet v 1 related allergen | gi\|281552896 |  | 22 | 314 | 617,3634 | 1232,7122 | K.ILEGDGCAGTIK.E + Carbamidomethyl (C) |
|  |  |  |  |  |  | 618,3832 | 1234,7518 | K.ILEGDGCAGTIK.E + Carbamidomethyl (C) |
|  |  |  |  |  |  | 638,8538 | 1275,6930 | K.IVACPDGGSICK.N + 2 Carbamidomethyl (C) |
|  |  |  |  |  |  | 638,8569 | 1275,6992 | K.IVACPDGGSICK.N + 2 Carbamidomethyl (C) |
|  |  |  |  |  |  | 638,8574 | 1275,7002 | K.IVACPDGGSICK.N + 2 Carbamidomethyl (C) |
|  |  |  |  |  |  | 639,3569 | 1276,6992 | K.IVACPDGGSICK.N + 2 Carbamidomethyl (C) |
|  |  |  |  |  |  | 644,8636 | 1287,7126 | K.AFILDGDTLVPK.V |
|  |  |  |  |  |  | 644,9109 | 1287,8072 | K.AFILDGDTLVPK.V |
| 4512 | Enolase | Achn086741 |  | 17 | 758 | 387,7216 | 773,4286 | K.ITGDQLK.D |
|  |  |  |  |  |  | 401,2395 | 800,4645 | K.EGLELLK.T |
|  |  |  |  |  |  | 403,7297 | 805,4449 | K.YNQLLR.I |
|  |  |  |  |  |  | 451,7602 | 901,5058 | K.ACNALLLK.E |
|  |  |  |  |  |  | 456,2080 | 910,4015 | R.DGGSDYLGK.G |
|  |  |  |  |  |  | 483,7408 | 965,4670 | K.MTCEIGKK.V |
|  |  |  |  |  |  | 322,8300 | 965,4681 | K.MTCEIGKK.V |
|  |  |  |  |  |  | 491,7379 | 981,4612 | K.MTCEIGKK.V |
|  |  |  |  |  |  | 630,8511 | 1259,6877 | K.AGASVQNIPLYK.E |
|  |  |  |  |  |  | 755,9282 | 1509,8419 | K.VQIVGDDLLVTNPK.R |
|  | Aspartate aminotransferase | Achn186891 |  | 15 | 417 | 301,7031 | 601,3916 | R.IKSVR.Q |
|  |  |  |  |  |  | 341,6818 | 681,3490 | R.YFPGAK.V |
|  |  |  |  |  |  | 425,2144 | 848,4143 | K.NIFNDAR.V |
|  |  |  |  |  |  | 447,7503 | 893,4861 | K.LFDSLTAK.D |
|  |  |  |  |  |  | 458,2609 | 914,5072 | K.IADVIQEK.N |
|  |  |  |  |  |  | 468,7325 | 935,4505 | R.VPWSEYR.Y |
|  |  |  |  |  |  | 473,7818 | 945,5490 | R.ISLAGLSSAK.C |
|  |  |  |  |  |  | 673,3755 | 1344,7365 | R.VATVQGLSGTGSLR.L |
| 4513 | Monodehydroascorbate reductase | gi\|284437984 |  | 7 | 373 | 336,2264 | 670,4383 | K.GIQIIK.G |
|  |  |  |  |  |  | 619,3068 | 1236,5991 | R.LTDFGVQGADSK.N |
| 4514 | Enolase | Achn354501 |  | 5 | 251 | 360,2004 | 718,3863 | K.ISGDSLK.N |
|  |  |  |  |  |  | 403,7299 | 805,4452 | K.YNQLLR.I |
|  |  |  |  |  |  | 456,2090 | 910,4034 | R.DGGSDYLGK.G |
|  |  |  |  |  |  | 459,7577 | 917,5008 | K.SCNALLLK.V |
| 4707 | Phosphoenolpyruvate carboxykinase | gi\|195203610 | 92% to gi\|220938756 Spartina maritima, E value:3e-93 | 16 | 112 | 675,4106 | 1348,8066 | R.AAYPIEYIPNAK.I |
|  |  |  |  |  |  | 807,9126 | 1613,8106 | R.DVDYSDNSVTENTR.A |
| 4708 | Mitochondrial-processing peptidase | Achn043531 |  | 10 | 349 | 308,1952 | 614,3759 | R.DVILR.E |
|  |  |  |  |  |  | 365,2223 | 728,4300 | R.ARNQLK.S |
|  |  |  |  |  |  | 380,2033 | 758,3921 | R.IDAVDAR.T |
|  |  |  |  |  |  | 420,7143 | 839,4141 | R.LENPDPR.F |
|  |  |  |  |  |  | 425,7428 | 849,4710 | R.QLITYGR.R |
|  |  |  |  |  |  | 438,7250 | 875,4355 | R.VSEADVTR.A |
|  |  |  |  |  |  | 509,7797 | 1017,5448 | R.IATESSLAAR.T |
|  |  |  |  |  |  | 542,3109 | 1082,6073 | R.TILGPAENIR.K |
| 4709 | Delta-1-pyrroline-5-carboxylate dehydrogenase | gi\|149938952 |  | 19 | 1300 | 329,7104 | 657,4062 | K.LAVDLK.G |
|  |  |  |  |  |  | 423,2582 | 844,5017 | K.LLQISGSK.L |
|  |  |  |  |  |  | 456,2690 | 910,5234 | K.LLLEGNPR.M |
|  |  |  |  |  |  | 459,7267 | 917,4388 | R.QMNDLAAR.R |
|  |  |  |  |  |  | 467,7236 | 933,4327 | R.QMNDLAAR.R |
|  |  |  |  |  |  | 314,2024 | 939,5854 | R.LIQRVSPK.S |
|  |  |  |  |  |  | 500,2497 | 998,4848 | R.MTLFTGSSR.V |
|  |  |  |  |  |  | 507,2849 | 1012,5553 | R.GAGIGTPEAIK.L |
|  |  |  |  |  |  | 508,2474 | 1014,4802 | R.MTLFTGSSR.V |
|  |  |  |  |  |  | 562,2724 | 1122,5302 | K.DNNYELVTR.E |
|  |  |  |  |  |  | 570,8036 | 1139,5927 | R.YLMLGDVSVK.A |
|  |  |  |  |  |  | 692,8199 | 1383,6252 | K.FLENFCGDQVR.F |
|  |  |  |  |  |  | 1017,5204 | 2033,0262 | K.VAEIDETEIQPFVESLSK.V |
| 4710 | Pyruvate decarboxylase | gi\|51587336 |  | 2 | 83 | 596,8517 | 1191,6888 | R.VAAANGRPPNPQ.- |
|  |  |  |  |  |  | 596,8544 | 1191,6942 | R.VAAANGRPPNPQ.- |
|  |  |  |  |  |  | 596,8545 | 1191,6944 | R.VAAANGRPPNPQ.- |
|  |  |  |  |  |  | 596,8550 | 1191,6954 | R.VAAANGRPPNPQ.- |
|  |  |  |  |  |  | 596,8563 | 1191,6980 | R.VAAANGRPPNPQ.- |
|  |  |  |  |  |  | 596,8568 | 1191,6990 | R.VAAANGRPPNPQ.- |
| 5405 | Transketolase | gi\|195315378 | 94% to gi\|110224784 Platanus x acerifolia, E value:2e-137 | 20 | 49 | 1027,4941 | 4105,9475 | R.FGVREHGMGAICNGIALHSPGLIPYCATFFVFTDYMR.A + Oxidation (M) |
|  |  |  |  |  |  | 1027,4959 | 4105,9543 | R.FGVREHGMGAICNGIALHSPGLIPYCATFFVFTDYMR.A + Oxidation (M) |
|  |  |  |  |  |  | 1027,4965 | 4105,9567 | R.FGVREHGMGAICNGIALHSPGLIPYCATFFVFTDYMR.A + Oxidation (M) |
|  |  |  |  |  |  | 1027,4977 | 4105,9616 | R.FGVREHGMGAICNGIALHSPGLIPYCATFFVFTDYMR.A + Oxidation (M) |
|  |  |  |  |  |  | 1027,4985 | 4105,9651 | R.FGVREHGMGAICNGIALHSPGLIPYCATFFVFTDYMR.A + Oxidation (M) |
|  |  |  |  |  |  | 1379,3162 | 4134,9267 | R.EHGMGAICNGIALHSPGLIPYCATFFVFTDYMRAAMR.I + Carbamidomethyl (C); Oxidation (M) |
|  |  |  |  |  |  | 1034,7393 | 4134,9279 | R.EHGMGAICNGIALHSPGLIPYCATFFVFTDYMRAAMR.I + Carbamidomethyl (C); Oxidation (M) |
|  |  |  |  |  |  | 1034,7407 | 4134,9338 | R.EHGMGAICNGIALHSPGLIPYCATFFVFTDYMRAAMR.I + Carbamidomethyl (C); Oxidation (M) |
|  |  |  |  |  |  | 1034,7407 | 4134,9338 | R.EHGMGAICNGIALHSPGLIPYCATFFVFTDYMRAAMR.I + Carbamidomethyl (C); Oxidation (M) |
|  |  |  |  |  |  | 1034,7411 | 4134,9353 | R.EHGMGAICNGIALHSPGLIPYCATFFVFTDYMRAAMR.I + Carbamidomethyl (C); Oxidation (M) |
|  |  |  |  |  |  | 1034,7412 | 4134,9357 | R.EHGMGAICNGIALHSPGLIPYCATFFVFTDYMRAAMR.I + Carbamidomethyl (C); Oxidation (M) |
|  |  |  |  |  |  | 1034,7418 | 4134,9382 | R.EHGMGAICNGIALHSPGLIPYCATFFVFTDYMRAAMR.I + Carbamidomethyl (C); Oxidation (M) |
|  |  |  |  |  |  | 1034,7419 | 4134,9387 | R.EHGMGAICNGIALHSPGLIPYCATFFVFTDYMRAAMR.I + Carbamidomethyl (C); Oxidation (M) |
|  |  |  |  |  |  | 1379,3237 | 4134,9494 | R.EHGMGAICNGIALHSPGLIPYCATFFVFTDYMRAAMR.I + Carbamidomethyl (C); Oxidation (M) |
| 5407 | GDP-mannose 4,6-dehydratase | Achn053211 |  | 3 | 31 | 350,7213 | 699,4281 | R.LLEAVR.S |
|  |  |  |  |  |  | 362,1870 | 722,3593 | R.SPYAASK.C |
|  |  |  |  |  |  | 362,1872 | 722,3598 | R.SPYAASK.C |
| 5712 | Non-identified |  |  |  |  |  |  |  |
| 5713 | Phosphoenolpyruvate carboxykinase | Achn041831 |  | 3 | 408 | 312,1800 | 622,3454 | K.LAYTR.K |
|  |  |  |  |  |  | 348,8403 | 1043,4992 | K.TTLSTDHNR.Y |
|  |  |  |  |  |  | 348,8403 | 1043,4992 | K.TTLSTDHNR.Y |
|  |  |  |  |  |  | 348,8404 | 1043,4993 | K.TTLSTDHNR.Y |
|  |  |  |  |  |  | 348,8405 | 1043,4996 | K.TTLSTDHNR.Y |
|  |  |  |  |  |  | 348,8407 | 1043,5002 | K.TTLSTDHNR.Y |
|  |  |  |  |  |  | 807,8467 | 1613,6789 | R.DVDYSDNSVTENTR.A |
|  |  |  |  |  |  | 807,8472 | 1613,6799 | R.DVDYSDNSVTENTR.A |
|  |  |  |  |  |  | 807,8475 | 1613,6805 | R.DVDYSDNSVTENTR.A |
|  |  |  |  |  |  | 807,8476 | 1613,6806 | R.DVDYSDNSVTENTR.A |
|  |  |  |  |  |  | 807,8478 | 1613,6811 | R.DVDYSDNSVTENTR.A |
|  |  |  |  |  |  | 807,8479 | 1613,6812 | R.DVDYSDNSVTENTR.A |
| 5808 | Pyruvate decarboxylase | Achn219321 |  | 4 | 208 | 436,7093 | 871,4041 | R.ENDEPLR.V |
|  |  |  |  |  |  | 448,7693 | 895,5240 | K.AVIVQPNR.V |
|  |  |  |  |  |  | 619,3166 | 1236,6187 | R.VSAANSRPPNPQ.- |
|  |  |  |  |  |  | 619,3167 | 1236,6189 | R.VSAANSRPPNPQ.- |
|  |  |  |  |  |  | 619,3175 | 1236,6204 | R.VSAANSRPPNPQ.- |
|  |  |  |  |  |  | 619,3178 | 1236,6209 | R.VSAANSRPPNPQ.- |
|  |  |  |  |  |  | 619,3179 | 1236,6212 | R.VSAANSRPPNPQ.- |
|  |  |  |  |  |  | 619,3179 | 1236,6213 | R.VSAANSRPPNPQ.- |
|  |  |  |  |  |  | 619,3179 | 1236,6213 | R.VSAANSRPPNPQ.- |
|  |  |  |  |  |  | 619,3180 | 1236,6215 | R.VSAANSRPPNPQ.- |
|  |  |  |  |  |  | 619,3183 | 1236,6220 | R.VSAANSRPPNPQ.- |
| 5907 | Alpha-glucosidase | Achn221901 |  | 1 | 104 | 345,2007 | 688,3868 | K.SVAVSAR.N |
|  |  |  |  |  |  | 345,2007 | 688,3869 | K.SVAVSAR.N |
|  |  |  |  |  |  | 345,2007 | 688,3869 | K.SVAVSAR.N |
|  |  |  |  |  |  | 345,2007 | 688,3869 | K.SVAVSAR.N |
|  |  |  |  |  |  | 345,2007 | 688,3869 | K.SVAVSAR.N |
|  |  |  |  |  |  | 345,2008 | 688,3871 | K.SVAVSAR.N |
|  |  |  |  |  |  | 378,2002 | 754,3859 | R.GSSLTYK.V |
|  |  |  |  |  |  | 378,2003 | 754,3860 | R.GSSLTYK.V |
|  |  |  |  |  |  | 378,2004 | 754,3863 | R.GSSLTYK.V |
|  |  |  |  |  |  | 378,2005 | 754,3864 | R.GSSLTYK.V |
|  |  |  |  |  |  | 378,2005 | 754,3864 | R.GSSLTYK.V |
|  |  |  |  |  |  | 378,2009 | 754,3872 | R.GSSLTYK.V |
| 5909 | Eukaryotic translation elongation factor | Achn004851 |  | 3 | 111 | 337,1847 | 672,3548 | R.QALGER.I |
|  |  |  |  |  |  | 352,6916 | 703,3686 | R.VAVQCK.V + Carbamidomethyl (C) |
|  |  |  |  |  |  | 352,6916 | 703,3687 | R.VAVQCK.V + Carbamidomethyl (C) |
|  |  |  |  |  |  | 352,6916 | 703,3687 | R.VAVQCK.V + Carbamidomethyl (C) |
|  |  |  |  |  |  | 352,6918 | 703,3690 | R.VAVQCK.V + Carbamidomethyl (C) |
|  |  |  |  |  |  | 352,6921 | 703,3697 | R.VAVQCK.V + Carbamidomethyl (C) |
|  |  |  |  |  |  | 359,7022 | 717,3898 | R.ETVLEK.S |
|  |  |  |  |  |  | 359,7027 | 717,3908 | R.ETVLEK.S |
|  |  |  |  |  |  | 359,7030 | 717,3914 | R.ETVLEK.S |
|  |  |  |  |  |  | 365,2104 | 728,4063 | K.VASDLPK.L |
|  |  |  |  |  |  | 365,2105 | 728,4065 | K.VASDLPK.L |
|  |  |  |  |  |  | 365,2110 | 728,4074 | K.VASDLPK.L |
|  |  |  |  |  |  | 365,2119 | 728,4091 | K.VASDLPK.L |
|  |  |  |  |  |  | 478,2695 | 954,5245 | R.GGGQVIPTAR.R |
|  |  |  |  |  |  | 478,2699 | 954,5252 | R.GGGQVIPTAR.R |
|  |  |  |  |  |  | 478,2706 | 954,5266 | R.GGGQVIPTAR.R |
| 5921 | Uncharacterized protein 2 | Achn042731 |  | 0 | 126 | 376,6815 | 751,3485 | K.DGEIYR.L |
|  |  |  |  |  |  | 376,6818 | 751,3490 | K.DGEIYR.L |
|  |  |  |  |  |  | 376,6823 | 751,3501 | K.DGEIYR.L |
|  |  |  |  |  |  | 376,6824 | 751,3502 | K.DGEIYR.L |
|  |  |  |  |  |  | 376,6824 | 751,3502 | K.DGEIYR.L |
|  |  |  |  |  |  | 376,6825 | 751,3505 | K.DGEIYR.L |
|  |  |  |  |  |  | 376,6826 | 751,3506 | K.DGEIYR.L |
| 6509 | Non-identified |  |  |  |  |  |  |  |
| 6604 | UDP-glucose dehydrogenase | Achn256641 |  | 7 | 173 | 315,6946 | 629,3746 | K.STVPVK.T |
|  |  |  |  |  |  | 315,6946 | 629,3747 | K.STVPVK.T |
|  |  |  |  |  |  | 315,6946 | 629,3747 | K.STVPVK.T |
|  |  |  |  |  |  | 315,6947 | 629,3748 | K.STVPVK.T |
|  |  |  |  |  |  | 315,6947 | 629,3748 | K.STVPVK.T |
|  |  |  |  |  |  | 315,6947 | 629,3749 | K.STVPVK.T |
|  |  |  |  |  |  | 315,6947 | 629,3749 | K.STVPVK.T |
|  |  |  |  |  |  | 322,2105 | 642,4065 | K.AIQALK.E |
|  |  |  |  |  |  | 322,2106 | 642,4066 | K.AIQALK.E |
|  |  |  |  |  |  | 381,2054 | 760,3963 | K.TAEAIEK.I |
|  |  |  |  |  |  | 381,2056 | 760,3966 | K.TAEAIEK.I |
|  |  |  |  |  |  | 381,2056 | 760,3967 | K.TAEAIEK.I |
|  |  |  |  |  |  | 381,2057 | 760,3968 | K.TAEAIEK.I |
|  |  |  |  |  |  | 381,2057 | 760,3969 | K.TAEAIEK.I |
|  |  |  |  |  |  | 381,2057 | 760,3969 | K.TAEAIEK.I |
|  |  |  |  |  |  | 381,2058 | 760,3969 | K.TAEAIEK.I |
|  |  |  |  |  |  | 381,2058 | 760,3971 | K.TAEAIEK.I |
|  |  |  |  |  |  | 516,7548 | 1031,4951 | R.ETPAIDVCK.G + Carbamidomethyl (C) |
|  |  |  |  |  |  | 516,7549 | 1031,4952 | R.ETPAIDVCK.G + Carbamidomethyl (C) |
|  |  |  |  |  |  | 516,7553 | 1031,4961 | R.ETPAIDVCK.G + Carbamidomethyl (C) |
|  |  |  |  |  |  | 516,7554 | 1031,4963 | R.ETPAIDVCK.G + Carbamidomethyl (C) |
|  |  |  |  |  |  | 516,7555 | 1031,4965 | R.ETPAIDVCK.G + Carbamidomethyl (C) |
| 6614 | Non-identified |  |  |  |  |  |  |  |
| 6708 | Natterin | gi\|195285924 | 70% to gi\|225465417 Vitis vinifera, E value:2e-92 | 11 | 72 | 675,3475 | 1348,6804 | K.TSCLNAAVSAISR.E + Carbamidomethyl (C) |
|  |  |  |  |  |  | 701,8086 | 1401,6026 | K.GSCDVPFSYSQR.D + Carbamidomethyl (C) |
| 6901 | Non-identified |  |  |  |  |  |  |  |
| 6946 | Kiwellin | Achn107521 |  | 3 | 94 | 490,2109 | 978,4072 | K.VVDECDSR.N + Carbamidomethyl (C) |
|  |  |  |  |  |  | 490,2109 | 978,4073 | K.VVDECDSR.N + Carbamidomethyl (C) |
|  |  |  |  |  |  | 490,2111 | 978,4076 | K.VVDECDSR.N + Carbamidomethyl (C) |
|  |  |  |  |  |  | 490,2112 | 978,4079 | K.VVDECDSR.N + Carbamidomethyl (C) |
| 7211 | Actinidin | gi\|195214977 | 76% to gi\|146215980 Actinidia deliciosa, E value:3e-92 | 17 | 415 | 322,2105 | 642,4064 | K.GAVVGIK.D |
|  |  |  |  |  |  | 322,2105 | 642,4065 | K.GAVVGIK.D |
|  |  |  |  |  |  | 322,2105 | 642,4065 | K.GAVVGIK.D |
|  |  |  |  |  |  | 322,2105 | 642,4065 | K.GAVVGIK.D |
|  |  |  |  |  |  | 322,2106 | 642,4066 | K.GAVVGIK.D |
|  |  |  |  |  |  | 322,2106 | 642,4067 | K.GAVVGIK.D |
|  |  |  |  |  |  | 492,7076 | 983,4006 | K.DGQCDVYK.K + Carbamidomethyl (C) |
|  |  |  |  |  |  | 492,7080 | 983,4015 | K.DGQCDVYK.K + Carbamidomethyl (C) |
|  |  |  |  |  |  | 492,7082 | 983,4018 | K.DGQCDVYK.K + Carbamidomethyl (C) |
|  |  |  |  |  |  | 492,7083 | 983,4020 | K.DGQCDVYK.K + Carbamidomethyl (C) |
|  |  |  |  |  |  | 492,7084 | 983,4022 | K.DGQCDVYK.K + Carbamidomethyl (C) |
|  |  |  |  |  |  | 556,7546 | 1111,4946 | K.DGQCDVYKK.N + Carbamidomethyl (C) |
|  |  |  |  |  |  | 772,4019 | 1542,7893 | K.AVASQPVSVGIDADSK.G |
|  |  |  |  |  |  | 772,4020 | 1542,7895 | K.AVASQPVSVGIDADSK.G |
|  |  |  |  |  |  | 772,4021 | 1542,7896 | K.AVASQPVSVGIDADSK.G |
|  |  |  |  |  |  | 772,4022 | 1542,7898 | K.AVASQPVSVGIDADSK.G |
|  |  |  |  |  |  | 772,4023 | 1542,7900 | K.AVASQPVSVGIDADSK.G |
|  |  |  |  |  |  | 772,4024 | 1542,7902 | K.AVASQPVSVGIDADSK.G |
| 7212 | 2-oxoglutarate dehydrogenase | Achn007391 |  | 1 | 104 | 579,7585 | 1157,5024 | R.SSQYCTDVAK.A + Carbamidomethyl (C) |
|  |  |  |  |  |  | 579,7585 | 1157,5025 | R.SSQYCTDVAK.A + Carbamidomethyl (C) |
|  |  |  |  |  |  | 579,7587 | 1157,5028 | R.SSQYCTDVAK.A + Carbamidomethyl (C) |
| 7607 | Natterin | gi\|195285924 | 70% to gi\|225465417 Vitis vinifera, E value:2e-92 | 37 | 843 | 366,2371 | 730,4597 | R.VSLLATK.G |
|  |  |  |  |  |  | 520,2697 | 1038,5249 | R.NIYNVNFR.L |
|  |  |  |  |  |  | 540,7485 | 1079,4824 | R.NLGNNNFCK.R |
|  |  |  |  |  |  | 581,7750 | 1161,5354 | R.SQEPNTIDMK.L |
|  |  |  |  |  |  | 589,7723 | 1177,5301 | R.SQEPNTIDMK.L |
|  |  |  |  |  |  | 618,3100 | 1234,6054 | R.MEVCELVISR.N |
|  |  |  |  |  |  | 412,8686 | 1235,5839 | R.NLGNNNFCKR.L |
|  |  |  |  |  |  | 618,7993 | 1235,5840 | R.NLGNNNFCKR.L |
|  |  |  |  |  |  | 626,3079 | 1250,6012 | R.MEVCELVISR.N |
|  |  |  |  |  |  | 675,3464 | 1348,6783 | K.TSCLNAAVSAISR.E |
|  |  |  |  |  |  | 700,3941 | 1398,7736 | K.TSVQTGIPLIADGK.V |
|  |  |  |  |  |  | 701,8068 | 1401,5990 | K.GSCDVPFSYSQR.D |
|  | NADP-dependent malic enzyme | Achn312431 |  | 9 | 308 | 383,2106 | 764,4066 | K.GLAFTEK.E |
|  |  |  |  |  |  | 395,6962 | 789,3778 | R.DSHYLR.G |
|  |  |  |  |  |  | 408,2163 | 814,4181 | K.APLEETR.K |
|  |  |  |  |  |  | 502,7744 | 1003,5343 | R.QYEVPLQK.Y |
|  |  |  |  |  |  | 508,7802 | 1015,5459 | R.KESLQHFK.K |
|  |  |  |  |  |  | 490,3080 | 1467,9020 | K.VIKPTVLIGTSGVGK.T |
|  |  |  |  |  |  | 734,9593 | 1467,9040 | K.VIKPTVLIGTSGVGK.T |
| 7608 | Natterin | Achn294821 |  | 12 | 548 | 303,2051 | 604,3955 | R.FVVLK.S |
|  |  |  |  |  |  | 303,2051 | 604,3956 | R.FVVLK.S |
|  |  |  |  |  |  | 303,2051 | 604,3956 | R.FVVLK.S |
|  |  |  |  |  |  | 303,2052 | 604,3958 | R.FVVLK.S |
|  |  |  |  |  |  | 303,2052 | 604,3958 | R.FVVLK.S |
|  |  |  |  |  |  | 366,2371 | 730,4596 | K.VSLLATK.G |
|  |  |  |  |  |  | 366,2371 | 730,4596 | K.VSLLATK.G |
|  |  |  |  |  |  | 366,2371 | 730,4597 | K.VSLLATK.G |
|  |  |  |  |  |  | 366,2371 | 730,4597 | K.VSLLATK.G |
|  |  |  |  |  |  | 366,2372 | 730,4598 | K.VSLLATK.G |
|  |  |  |  |  |  | 366,2372 | 730,4599 | K.VSLLATK.G |
|  |  |  |  |  |  | 366,2373 | 730,4600 | K.VSLLATK.G |
|  |  |  |  |  |  | 366,2373 | 730,4600 | K.VSLLATK.G |
|  |  |  |  |  |  | 402,7324 | 803,4502 | K.RLTTEGK.T |
|  |  |  |  |  |  | 402,7325 | 803,4505 | K.RLTTEGK.T |
|  |  |  |  |  |  | 402,7326 | 803,4506 | K.RLTTEGK.T |
|  |  |  |  |  |  | 507,2904 | 1012,5663 | K.VDNNIVALR.N |
|  |  |  |  |  |  | 507,2904 | 1012,5663 | K.VDNNIVALR.N |
|  |  |  |  |  |  | 507,2909 | 1012,5672 | K.VDNNIVALR.N |
|  |  |  |  |  |  | 540,7487 | 1079,4828 | R.NLGNNNFCK.R + Carbamidomethyl (C) |
|  |  |  |  |  |  | 581,7739 | 1161,5332 | R.SQEPNTIDMK.L |
|  |  |  |  |  |  | 581,7744 | 1161,5341 | R.SQEPNTIDMK.L |
|  |  |  |  |  |  | 581,7747 | 1161,5348 | R.SQEPNTIDMK.L |
|  |  |  |  |  |  | 581,7747 | 1161,5348 | R.SQEPNTIDMK.L |
|  |  |  |  |  |  | 581,7752 | 1161,5359 | R.SQEPNTIDMK.L |
|  |  |  |  |  |  | 589,7714 | 1177,5282 | R.SQEPNTIDMK.L + Oxidation (M) |
|  |  |  |  |  |  | 589,7715 | 1177,5284 | R.SQEPNTIDMK.L + Oxidation (M) |
|  |  |  |  |  |  | 589,7719 | 1177,5293 | R.SQEPNTIDMK.L + Oxidation (M) |
|  |  |  |  |  |  | 589,7720 | 1177,5294 | R.SQEPNTIDMK.L + Oxidation (M) |
|  |  |  |  |  |  | 589,7720 | 1177,5295 | R.SQEPNTIDMK.L + Oxidation (M) |
|  |  |  |  |  |  | 589,7722 | 1177,5299 | R.SQEPNTIDMK.L + Oxidation (M) |
|  |  |  |  |  |  | 589,7723 | 1177,5300 | R.SQEPNTIDMK.L + Oxidation (M) |
|  |  |  |  |  |  | 701,8059 | 1401,5973 | K.GSCDVPFSYSQR.D + Carbamidomethyl (C) |
| 7609 | Pectinesterase | gi\|160419153 |  | 14 | 784 | 397,7352 | 793,4558 | K.HQAVALR.S |
|  |  |  |  |  |  | 398,6922 | 795,3698 | K.CNLYAR.K |
|  |  |  |  |  |  | 453,7433 | 905,4720 | K.NIFTAQGR.D |
|  |  |  |  |  |  | 635,3093 | 1268,6040 | R.GISFENYAGPSK.H |
|  |  |  |  |  |  | 844,8998 | 1687,7850 | R.DDPNQNTGISILNCK.V |
| 7709 | Natterin | gi\|195285924 | 70% to gi\|225465417 Vitis vinifera, E value:2e-92 | 15 | 108 | 581,7766 | 1161,5386 | R.SQEPNTIDMK.L |
|  |  |  |  |  |  | 675,3476 | 1348,6806 | K.TSCLNAAVSAISR.E + Carbamidomethyl (C) |
|  |  |  |  |  |  | 701,8047 | 1401,5948 | K.GSCDVPFSYSQR.D + Carbamidomethyl (C) |
|  |  |  |  |  |  | 701,8074 | 1401,6002 | K.GSCDVPFSYSQR.D + Carbamidomethyl (C) |
| 7712 | Non-identified |  |  |  |  |  |  |  |
| 7714 | NADP-dependent malic enzyme | Achn312431 |  | 2 | 89 | 386,7323 | 771,4500 | K.DLLNAVK.V |
|  |  |  |  |  |  | 408,2166 | 814,4187 | K.APLEETR.K |
|  |  |  |  |  |  | 408,2166 | 814,4187 | K.APLEETR.K |
|  |  |  |  |  |  | 408,2166 | 814,4187 | K.APLEETR.K |
|  |  |  |  |  |  | 408,2166 | 814,4187 | K.APLEETR.K |
|  |  |  |  |  |  | 408,2166 | 814,4187 | K.APLEETR.K |
|  |  |  |  |  |  | 408,2167 | 814,4189 | K.APLEETR.K |
| 7715 | Remorin | Achn227731 |  | 1 | 70 | 417,2093 | 832,4041 | K.DAASNISR.A |
|  |  |  |  |  |  | 417,2093 | 832,4041 | K.DAASNISR.A |
|  |  |  |  |  |  | 417,2093 | 832,4041 | K.DAASNISR.A |
|  |  |  |  |  |  | 417,2093 | 832,4041 | K.DAASNISR.A |
|  |  |  |  |  |  | 417,2094 | 832,4042 | K.DAASNISR.A |
|  |  |  |  |  |  | 417,2094 | 832,4043 | K.DAASNISR.A |
|  |  |  |  |  |  | 417,2095 | 832,4044 | K.DAASNISR.A |
|  |  |  |  |  |  | 417,2095 | 832,4044 | K.DAASNISR.A |
|  |  |  |  |  |  | 417,2095 | 832,4044 | K.DAASNISR.A |
|  |  |  |  |  |  | 417,2096 | 832,4046 | K.DAASNISR.A |
|  |  |  |  |  |  | 417,2096 | 832,4046 | K.DAASNISR.A |
|  |  |  |  |  |  | 417,2096 | 832,4047 | K.DAASNISR.A |
| 7720 | Natterin | Achn294821 |  | 7 | 568 | 303,2049 | 604,3952 | R.FVVLK.S |
|  |  |  |  |  |  | 303,2049 | 604,3952 | R.FVVLK.S |
|  |  |  |  |  |  | 303,2049 | 604,3953 | R.FVVLK.S |
|  |  |  |  |  |  | 303,2050 | 604,3954 | R.FVVLK.S |
|  |  |  |  |  |  | 303,2050 | 604,3954 | R.FVVLK.S |
|  |  |  |  |  |  | 303,2050 | 604,3955 | R.FVVLK.S |
|  |  |  |  |  |  | 324,6817 | 647,3488 | R.LTTEGK.T |
|  |  |  |  |  |  | 324,6817 | 647,3488 | R.LTTEGK.T |
|  |  |  |  |  |  | 324,6817 | 647,3489 | R.LTTEGK.T |
|  |  |  |  |  |  | 324,6818 | 647,3491 | R.LTTEGK.T |
|  |  |  |  |  |  | 324,6818 | 647,3491 | R.LTTEGK.T |
|  |  |  |  |  |  | 324,6819 | 647,3492 | R.LTTEGK.T |
|  |  |  |  |  |  | 324,6819 | 647,3493 | R.LTTEGK.T |
|  |  |  |  |  |  | 324,6820 | 647,3495 | R.LTTEGK.T |
|  |  |  |  |  |  | 324,6820 | 647,3495 | R.LTTEGK.T |
|  |  |  |  |  |  | 324,6821 | 647,3496 | R.LTTEGK.T |
|  |  |  |  |  |  | 324,6822 | 647,3498 | R.LTTEGK.T |
|  |  |  |  |  |  | 366,2367 | 730,4589 | K.VSLLATK.G |
|  |  |  |  |  |  | 366,2368 | 730,4590 | K.VSLLATK.G |
|  |  |  |  |  |  | 366,2368 | 730,4590 | K.VSLLATK.G |
|  |  |  |  |  |  | 366,2369 | 730,4591 | K.VSLLATK.G |
|  |  |  |  |  |  | 366,2369 | 730,4593 | K.VSLLATK.G |
|  |  |  |  |  |  | 366,2369 | 730,4593 | K.VSLLATK.G |
|  |  |  |  |  |  | 366,2369 | 730,4593 | K.VSLLATK.G |
|  |  |  |  |  |  | 366,2371 | 730,4596 | K.VSLLATK.G |
|  |  |  |  |  |  | 428,2138 | 854,4129 | K.LSYTDTR.S |
|  |  |  |  |  |  | 428,2138 | 854,4130 | K.LSYTDTR.S |
|  |  |  |  |  |  | 428,2139 | 854,4132 | K.LSYTDTR.S |
|  |  |  |  |  |  | 428,2140 | 854,4134 | K.LSYTDTR.S |
|  |  |  |  |  |  | 428,2140 | 854,4135 | K.LSYTDTR.S |
|  |  |  |  |  |  | 428,2140 | 854,4135 | K.LSYTDTR.S |
|  |  |  |  |  |  | 581,7734 | 1161,5323 | R.SQEPNTIDMK.L |
|  |  |  |  |  |  | 581,7740 | 1161,5335 | R.SQEPNTIDMK.L |
|  |  |  |  |  |  | 581,7741 | 1161,5337 | R.SQEPNTIDMK.L |
|  |  |  |  |  |  | 581,7743 | 1161,5340 | R.SQEPNTIDMK.L |
|  |  |  |  |  |  | 581,7746 | 1161,5346 | R.SQEPNTIDMK.L |
|  |  |  |  |  |  | 581,7747 | 1161,5349 | R.SQEPNTIDMK.L |
|  |  |  |  |  |  | 589,7701 | 1177,5256 | R.SQEPNTIDMK.L + Oxidation (M) |
|  |  |  |  |  |  | 589,7715 | 1177,5284 | R.SQEPNTIDMK.L + Oxidation (M) |
|  |  |  |  |  |  | 589,7717 | 1177,5288 | R.SQEPNTIDMK.L + Oxidation (M) |
|  |  |  |  |  |  | 589,7717 | 1177,5288 | R.SQEPNTIDMK.L + Oxidation (M) |
|  |  |  |  |  |  | 589,7718 | 1177,5291 | R.SQEPNTIDMK.L + Oxidation (M) |
|  |  |  |  |  |  | 589,7719 | 1177,5293 | R.SQEPNTIDMK.L + Oxidation (M) |
|  |  |  |  |  |  | 589,7721 | 1177,5296 | R.SQEPNTIDMK.L + Oxidation (M) |
| 7802 | Remorin | Achn227731 |  | 1 | 58 | 417,2093 | 832,4040 | K.DAASNISR.A |
|  |  |  |  |  |  | 417,2093 | 832,4041 | K.DAASNISR.A |
|  |  |  |  |  |  | 417,2093 | 832,4041 | K.DAASNISR.A |
|  |  |  |  |  |  | 417,2094 | 832,4042 | K.DAASNISR.A |
|  |  |  |  |  |  | 417,2094 | 832,4043 | K.DAASNISR.A |
|  |  |  |  |  |  | 417,2094 | 832,4043 | K.DAASNISR.A |
|  |  |  |  |  |  | 417,2094 | 832,4043 | K.DAASNISR.A |
|  |  |  |  |  |  | 417,2094 | 832,4043 | K.DAASNISR.A |
|  |  |  |  |  |  | 417,2095 | 832,4045 | K.DAASNISR.A |
|  |  |  |  |  |  | 417,2096 | 832,4046 | K.DAASNISR.A |
|  |  |  |  |  |  | 417,2097 | 832,4048 | K.DAASNISR.A |
| 7901 | Beta-D-galactosidase | gi\|318136780 |  | 6 | 401 | 333,1844 | 664,3542 | R.YDLVR.F |
|  |  |  |  |  |  | 333,1845 | 664,3545 | R.YDLVR.F |
|  |  |  |  |  |  | 333,1849 | 664,3552 | R.YDLVR.F |
|  |  |  |  |  |  | 366,7163 | 731,4180 | R.DLTKQK.W |
|  |  |  |  |  |  | 366,7164 | 731,4183 | R.DLTKQK.W |
|  |  |  |  |  |  | 366,7165 | 731,4185 | R.DLTKQK.W |
|  |  |  |  |  |  | 373,6930 | 745,3715 | K.GLNEGTR.D |
|  |  |  |  |  |  | 373,6931 | 745,3717 | K.GLNEGTR.D |
|  |  |  |  |  |  | 373,6931 | 745,3717 | K.GLNEGTR.D |
|  |  |  |  |  |  | 373,6932 | 745,3717 | K.GLNEGTR.D |
|  |  |  |  |  |  | 373,6932 | 745,3717 | K.GLNEGTR.D |
|  |  |  |  |  |  | 373,6932 | 745,3718 | K.GLNEGTR.D |
|  |  |  |  |  |  | 373,6932 | 745,3719 | K.GLNEGTR.D |
|  |  |  |  |  |  | 641,2692 | 1280,5239 | K.CLSNCGEASQR.W + 2 Carbamidomethyl (C) |
|  |  |  |  |  |  | 641,2692 | 1280,5239 | K.CLSNCGEASQR.W + 2 Carbamidomethyl (C) |
|  |  |  |  |  |  | 641,2693 | 1280,5240 | K.CLSNCGEASQR.W + 2 Carbamidomethyl (C) |
|  |  |  |  |  |  | 641,2693 | 1280,5240 | K.CLSNCGEASQR.W + 2 Carbamidomethyl (C) |
|  |  |  |  |  |  | 641,2693 | 1280,5240 | K.CLSNCGEASQR.W + 2 Carbamidomethyl (C) |
|  |  |  |  |  |  | 641,2695 | 1280,5244 | K.CLSNCGEASQR.W + 2 Carbamidomethyl (C) |
|  |  |  |  |  |  | 641,2698 | 1280,5250 | K.CLSNCGEASQR.W + 2 Carbamidomethyl (C) |
|  |  |  |  |  |  | 897,8761 | 1793,7376 | K.ASGNCGGCSYAGIYTEK.K + 2 Carbamidomethyl (C) |
| 7902 | Beta-D-galactosidase | gi\|318136780 |  | 5 | 461 | 366,7163 | 731,4181 | R.DLTKQK.W |
|  |  |  |  |  |  | 373,6933 | 745,3720 | K.GLNEGTR.D |
|  |  |  |  |  |  | 373,6933 | 745,3720 | K.GLNEGTR.D |
|  |  |  |  |  |  | 373,6933 | 745,3721 | K.GLNEGTR.D |
|  |  |  |  |  |  | 373,6933 | 745,3721 | K.GLNEGTR.D |
|  |  |  |  |  |  | 373,6934 | 745,3722 | K.GLNEGTR.D |
|  |  |  |  |  |  | 373,6934 | 745,3722 | K.GLNEGTR.D |
|  |  |  |  |  |  | 641,2687 | 1280,5229 | K.CLSNCGEASQR.W + 2 Carbamidomethyl (C) |
|  |  |  |  |  |  | 641,2693 | 1280,5240 | K.CLSNCGEASQR.W + 2 Carbamidomethyl (C) |
|  |  |  |  |  |  | 641,2694 | 1280,5241 | K.CLSNCGEASQR.W + 2 Carbamidomethyl (C) |
|  |  |  |  |  |  | 641,2694 | 1280,5241 | K.CLSNCGEASQR.W + 2 Carbamidomethyl (C) |
|  |  |  |  |  |  | 641,2695 | 1280,5245 | K.CLSNCGEASQR.W + 2 Carbamidomethyl (C) |
|  |  |  |  |  |  | 897,8735 | 1793,7324 | K.ASGNCGGCSYAGIYTEK.K + 2 Carbamidomethyl (C) |
|  |  |  |  |  |  | 897,8736 | 1793,7326 | K.ASGNCGGCSYAGIYTEK.K + 2 Carbamidomethyl (C) |
|  |  |  |  |  |  | 897,8737 | 1793,7328 | K.ASGNCGGCSYAGIYTEK.K + 2 Carbamidomethyl (C) |
| 7903 | Beta-D-galactosidase | gi\|318136780 |  | 5 | 343 | 333,1846 | 664,3546 | R.YDLVR.F |
|  |  |  |  |  |  | 373,6932 | 745,3718 | K.GLNEGTR.D |
|  |  |  |  |  |  | 373,6932 | 745,3718 | K.GLNEGTR.D |
|  |  |  |  |  |  | 373,6932 | 745,3719 | K.GLNEGTR.D |
|  |  |  |  |  |  | 373,6932 | 745,3719 | K.GLNEGTR.D |
|  |  |  |  |  |  | 373,6933 | 745,3720 | K.GLNEGTR.D |
|  |  |  |  |  |  | 641,2679 | 1280,5213 | K.CLSNCGEASQR.W + 2 Carbamidomethyl (C) |
|  |  |  |  |  |  | 641,2690 | 1280,5234 | K.CLSNCGEASQR.W + 2 Carbamidomethyl (C) |
|  |  |  |  |  |  | 641,2692 | 1280,5239 | K.CLSNCGEASQR.W + 2 Carbamidomethyl (C) |
|  |  |  |  |  |  | 641,2692 | 1280,5239 | K.CLSNCGEASQR.W + 2 Carbamidomethyl (C) |
|  |  |  |  |  |  | 641,2695 | 1280,5244 | K.CLSNCGEASQR.W + 2 Carbamidomethyl (C) |
|  |  |  |  |  |  | 897,8737 | 1793,7329 | K.ASGNCGGCSYAGIYTEK.K + 2 Carbamidomethyl (C) |
|  |  |  |  |  |  | 897,8774 | 1793,7402 | K.ASGNCGGCSYAGIYTEK.K + 2 Carbamidomethyl (C) |
| 7904 | Beta-D-galactosidase | gi\|318136780 |  | 2 | 58 | 373,6933 | 745,3721 | K.GLNEGTR.D |
|  |  |  |  |  |  | 373,6934 | 745,3723 | K.GLNEGTR.D |
|  |  |  |  |  |  | 641,2679 | 1280,5213 | K.CLSNCGEASQR.W + 2 Carbamidomethyl (C) |
| 7907 | Beta-D-galactosidase | gi\|318136780 |  | 4 | 294 | 333,1846 | 664,3547 | R.YDLVR.F |
|  |  |  |  |  |  | 333,1848 | 664,3550 | R.YDLVR.F |
|  |  |  |  |  |  | 333,1848 | 664,3550 | R.YDLVR.F |
|  |  |  |  |  |  | 373,6931 | 745,3717 | K.GLNEGTR.D |
|  |  |  |  |  |  | 373,6931 | 745,3717 | K.GLNEGTR.D |
|  |  |  |  |  |  | 373,6932 | 745,3717 | K.GLNEGTR.D |
|  |  |  |  |  |  | 373,6932 | 745,3719 | K.GLNEGTR.D |
|  |  |  |  |  |  | 373,6932 | 745,3719 | K.GLNEGTR.D |
|  |  |  |  |  |  | 373,6933 | 745,3720 | K.GLNEGTR.D |
|  |  |  |  |  |  | 446,7354 | 891,4563 | K.NAVFNTAR.V |
|  |  |  |  |  |  | 641,2689 | 1280,5232 | K.CLSNCGEASQR.W + 2 Carbamidomethyl (C) |
|  |  |  |  |  |  | 641,2692 | 1280,5238 | K.CLSNCGEASQR.W + 2 Carbamidomethyl (C) |
|  |  |  |  |  |  | 641,2698 | 1280,5251 | K.CLSNCGEASQR.W + 2 Carbamidomethyl (C) |
|  |  |  |  |  |  | 641,2699 | 1280,5252 | K.CLSNCGEASQR.W + 2 Carbamidomethyl (C) |
|  |  |  |  |  |  | 641,2700 | 1280,5254 | K.CLSNCGEASQR.W + 2 Carbamidomethyl (C) |
|  |  |  |  |  |  | 641,2701 | 1280,5257 | K.CLSNCGEASQR.W + 2 Carbamidomethyl (C) |
| 8102 | Thaumatin | gi\|146737976 |  | 12 | 1395 | 527,7220 | 1053,4294 | R.TGCNFDGAGR.G |
|  |  |  |  |  |  | 903,3839 | 1804,7531 | K.DDQTSTFTCPAGTNYK.V |
| 8119 | Formate dehydrogenase | gi\|195211331 | 94% to gi\|225452472 Vitis vinifera, E value:5e-98 | 20 | 164 | 364,7552 | 727,4959 | R.ILILTR.N |
|  |  |  |  |  |  | 398,1977 | 794,3809 | R.AYDLEGK.T |
|  |  |  |  |  |  | 409,2299 | 816,4453 | K.TVGTVGAGR.I |
|  |  |  |  |  |  | 478,2879 | 954,5613 | K.GVLIVNNAR.G |
|  | Catalase | Achn051741 |  | 5 | 123 | 309,1744 | 616,3343 | K.AHYVK.F |
|  |  |  |  |  |  | 494,7640 | 987,5135 | R.FSTVIHER.G |
|  |  |  |  |  |  | 568,8431 | 1135,6716 | R.APGVQTPVIVR.F |
| 8415 | Uncharacterized protein 3 | gi\|195193220 | 86% to gi\|596001390 Prunus persica, E value:1e-70 | 12 | 74 | 344,2055 | 686,3964 | R.AIADVAK.L |
|  |  |  |  |  |  | 344,2056 | 686,3966 | R.AIADVAK.L |
|  |  |  |  |  |  | 344,2057 | 686,3968 | R.AIADVAK.L |
|  |  |  |  |  |  | 344,2057 | 686,3969 | R.AIADVAK.L |
|  |  |  |  |  |  | 553,2689 | 1104,5232 | R.TCAQEEVLR.V + Carbamidomethyl (C) |
|  |  |  |  |  |  | 553,2695 | 1104,5245 | R.TCAQEEVLR.V + Carbamidomethyl (C) |
| 8604 | RNase Phy3, partial | gi\|195320811 | 36% to gi\|258617486 Petunia x hybrida, E value:3e-19 | 31 | 360 | 402,2057 | 802,3969 | K.NWDQIK.K |
|  |  |  |  |  |  | 429,7557 | 857,4969 | K.SAVQNVIK.T |
|  |  |  |  |  |  | 466,2400 | 930,4654 | K.SGIVPDDTK.L |
|  |  |  |  |  |  | 484,2582 | 966,5018 | K.FETTTQLK.A |
|  |  |  |  |  |  | 556,8378 | 1111,6610 | K.LNNLLDVLAK.S |
|  |  |  |  |  |  | 662,2860 | 1322,5575 | K.YESCPASQEPR.G |
|  |  |  |  |  |  | 670,8296 | 1339,6446 | K.TNNVYISCLEK.N |
| 8605 | Elongation factor | gi\|61741088 |  | 4 | 194 | 513,3084 | 1024,6023 | K.IGGIGTVPVGR.V |
|  |  |  |  |  |  | 560,8028 | 1119,5910 | K.STTTGHLIYK.L |
| 8802 | Remorin | Achn227731 |  | 1 | 70 | 417,2090 | 832,4035 | K.DAASNISR.A |
|  |  |  |  |  |  | 417,2091 | 832,4037 | K.DAASNISR.A |
|  |  |  |  |  |  | 417,2092 | 832,4038 | K.DAASNISR.A |
|  |  |  |  |  |  | 417,2092 | 832,4039 | K.DAASNISR.A |
|  |  |  |  |  |  | 417,2093 | 832,4040 | K.DAASNISR.A |
|  |  |  |  |  |  | 417,2093 | 832,4040 | K.DAASNISR.A |
|  |  |  |  |  |  | 417,2093 | 832,4040 | K.DAASNISR.A |
|  |  |  |  |  |  | 417,2093 | 832,4041 | K.DAASNISR.A |
| 8804 | Beta-D-galactosidase | gi\|318136780 |  | 3 | 211 | 333,1845 | 664,3545 | R.YDLVR.F |
|  |  |  |  |  |  | 333,1847 | 664,3548 | R.YDLVR.F |
|  |  |  |  |  |  | 333,1848 | 664,3549 | R.YDLVR.F |
|  |  |  |  |  |  | 373,6931 | 745,3715 | K.GLNEGTR.D |
|  |  |  |  |  |  | 373,6931 | 745,3716 | K.GLNEGTR.D |
|  |  |  |  |  |  | 373,6931 | 745,3716 | K.GLNEGTR.D |
|  |  |  |  |  |  | 373,6931 | 745,3717 | K.GLNEGTR.D |
|  |  |  |  |  |  | 373,6932 | 745,3717 | K.GLNEGTR.D |
|  |  |  |  |  |  | 373,6932 | 745,3718 | K.GLNEGTR.D |
|  |  |  |  |  |  | 641,2690 | 1280,5235 | K.CLSNCGEASQR.W + 2 Carbamidomethyl (C) |
|  |  |  |  |  |  | 641,2693 | 1280,5240 | K.CLSNCGEASQR.W + 2 Carbamidomethyl (C) |
|  |  |  |  |  |  | 641,2695 | 1280,5245 | K.CLSNCGEASQR.W + 2 Carbamidomethyl (C) |
|  |  |  |  |  |  | 641,2702 | 1280,5258 | K.CLSNCGEASQR.W + 2 Carbamidomethyl (C) |
| 8805 | Quinohemoprotein ethanol dehydrogenase | gi\|195257582 | 76% to gi\|508728426 Theobroma cacao, E value:2e-65 | 11 | 240 | 415,2428 | 828,4711 | R.DVAVAVQK.S |
|  |  |  |  |  |  | 493,2929 | 984,5713 | K.RDVAVAVQK.S |
|  |  |  |  |  |  | 540,7607 | 1079,5069 | R.EGPVYAMSAR.S |
|  |  |  |  |  |  | 548,7584 | 1095,5023 | R.EGPVYAMSAR.S |
| 8902 | Beta-D-galactosidase | gi\|318136780 |  | 2 | 250 | 373,6931 | 745,3716 | K.GLNEGTR.D |
|  |  |  |  |  |  | 373,6933 | 745,3720 | K.GLNEGTR.D |
|  |  |  |  |  |  | 373,6933 | 745,3721 | K.GLNEGTR.D |
|  |  |  |  |  |  | 373,6934 | 745,3722 | K.GLNEGTR.D |
|  |  |  |  |  |  | 641,2687 | 1280,5229 | K.CLSNCGEASQR.W + 2 Carbamidomethyl (C) |
|  |  |  |  |  |  | 641,2691 | 1280,5236 | K.CLSNCGEASQR.W + 2 Carbamidomethyl (C) |
|  |  |  |  |  |  | 641,2693 | 1280,5240 | K.CLSNCGEASQR.W + 2 Carbamidomethyl (C) |
|  |  |  |  |  |  | 641,2695 | 1280,5244 | K.CLSNCGEASQR.W + 2 Carbamidomethyl (C) |
|  |  |  |  |  |  | 641,2702 | 1280,5258 | K.CLSNCGEASQR.W + 2 Carbamidomethyl (C) |
|  |  |  |  |  |  | 641,2703 | 1280,5261 | K.CLSNCGEASQR.W + 2 Carbamidomethyl (C) |
| 8903 | Quinohemoprotein ethanol dehydrogenase | gi\|195196846 | 68% to gi\|449520922 Cucumis sativus, E value:2e-69 | 7 | 136 | 378,1816 | 754,3487 | R.YASGETK.I |
|  |  |  |  |  |  | 378,1817 | 754,3488 | R.YASGETK.I |
|  |  |  |  |  |  | 378,1817 | 754,3488 | R.YASGETK.I |
|  |  |  |  |  |  | 378,1819 | 754,3493 | R.YASGETK.I |
|  |  |  |  |  |  | 378,1820 | 754,3495 | R.YASGETK.I |
|  |  |  |  |  |  | 378,1820 | 754,3495 | R.YASGETK.I |
|  |  |  |  |  |  | 378,1821 | 754,3497 | R.YASGETK.I |
|  |  |  |  |  |  | 378,1822 | 754,3498 | R.YASGETK.I |
|  |  |  |  |  |  | 378,1822 | 754,3498 | R.YASGETK.I |
|  |  |  |  |  |  | 378,1822 | 754,3498 | R.YASGETK.I |
|  |  |  |  |  |  | 378,1822 | 754,3499 | R.YASGETK.I |
|  |  |  |  |  |  | 378,1822 | 754,3499 | R.YASGETK.I |
|  |  |  |  |  |  | 378,1823 | 754,3500 | R.YASGETK.I |
|  |  |  |  |  |  | 378,1823 | 754,3500 | R.YASGETK.I |
|  |  |  |  |  |  | 378,1823 | 754,3501 | R.YASGETK.I |
|  |  |  |  |  |  | 378,1823 | 754,3501 | R.YASGETK.I |
|  |  |  |  |  |  | 378,1824 | 754,3502 | R.YASGETK.I |
|  |  |  |  |  |  | 378,1824 | 754,3503 | R.YASGETK.I |
|  |  |  |  |  |  | 378,1825 | 754,3504 | R.YASGETK.I |
|  |  |  |  |  |  | 378,1827 | 754,3507 | R.YASGETK.I |
|  |  |  |  |  |  | 378,1827 | 754,3509 | R.YASGETK.I |
|  |  |  |  |  |  | 378,1828 | 754,3510 | R.YASGETK.I |
|  |  |  |  |  |  | 378,1829 | 754,3512 | R.YASGETK.I |
|  |  |  |  |  |  | 415,2425 | 828,4704 | R.DVAVAVQK.S |
|  |  |  |  |  |  | 415,2425 | 828,4704 | R.DVAVAVQK.S |
|  |  |  |  |  |  | 415,2425 | 828,4705 | R.DVAVAVQK.S |
|  |  |  |  |  |  | 415,2426 | 828,4706 | R.DVAVAVQK.S |
|  |  |  |  |  |  | 415,2427 | 828,4709 | R.DVAVAVQK.S |
|  |  |  |  |  |  | 415,2428 | 828,4710 | R.DVAVAVQK.S |
|  |  |  |  |  |  | 415,2430 | 828,4715 | R.DVAVAVQK.S |
| 9106 | Thaumatin | gi\|190358875 |  | 44 | 26701 | 311,1570 | 620,2995 | K.VVFCP.- |
|  |  |  |  |  |  | 527,7225 | 1053,4304 | R.TGCNFDGAGR.G |
|  |  |  |  |  |  | 550,7394 | 1099,4643 | R.CPDAYSYPK.D |
|  |  |  |  |  |  | 678,3567 | 1354,6988 | R.GQNWIINPGAGTK.G |
|  |  |  |  |  |  | 711,3189 | 1420,6233 | R.APGGCNNPCTVFK.T |
|  |  |  |  |  |  | 824,8618 | 1647,7091 | K.CTADINGQCPNELR.A |
|  |  |  |  |  |  | 870,4631 | 1738,9116 | R.LDRGQNWIINPGAGTK.G |
|  |  |  |  |  |  | 580,6446 | 1738,9119 | R.LDRGQNWIINPGAGTK.G |
|  |  |  |  |  |  | 903,3866 | 1804,7586 | K.DDQTSTFTCPAGTNYK.V |
|  |  |  |  |  |  | 1063,4208 | 2124,8270 | K.TDQYCCNSGNCGLTNFSK.F |
|  |  |  |  |  |  | 709,2847 | 2124,8322 | K.TDQYCCNSGNCGLTNFSK.F |
