# Supplementary figures and images for: Coupling of Physiological and Proteomic Analysis to Understand the Ethylene- and Chilling-Induced Kiwifruit Ripening Syndrome
Source: Front Plant Sci. 2016 Feb 15;7:120. doi: 10.3389/fpls.2016.00120 (PMC4753329; doi:10.3389/fpls.2016.00120)

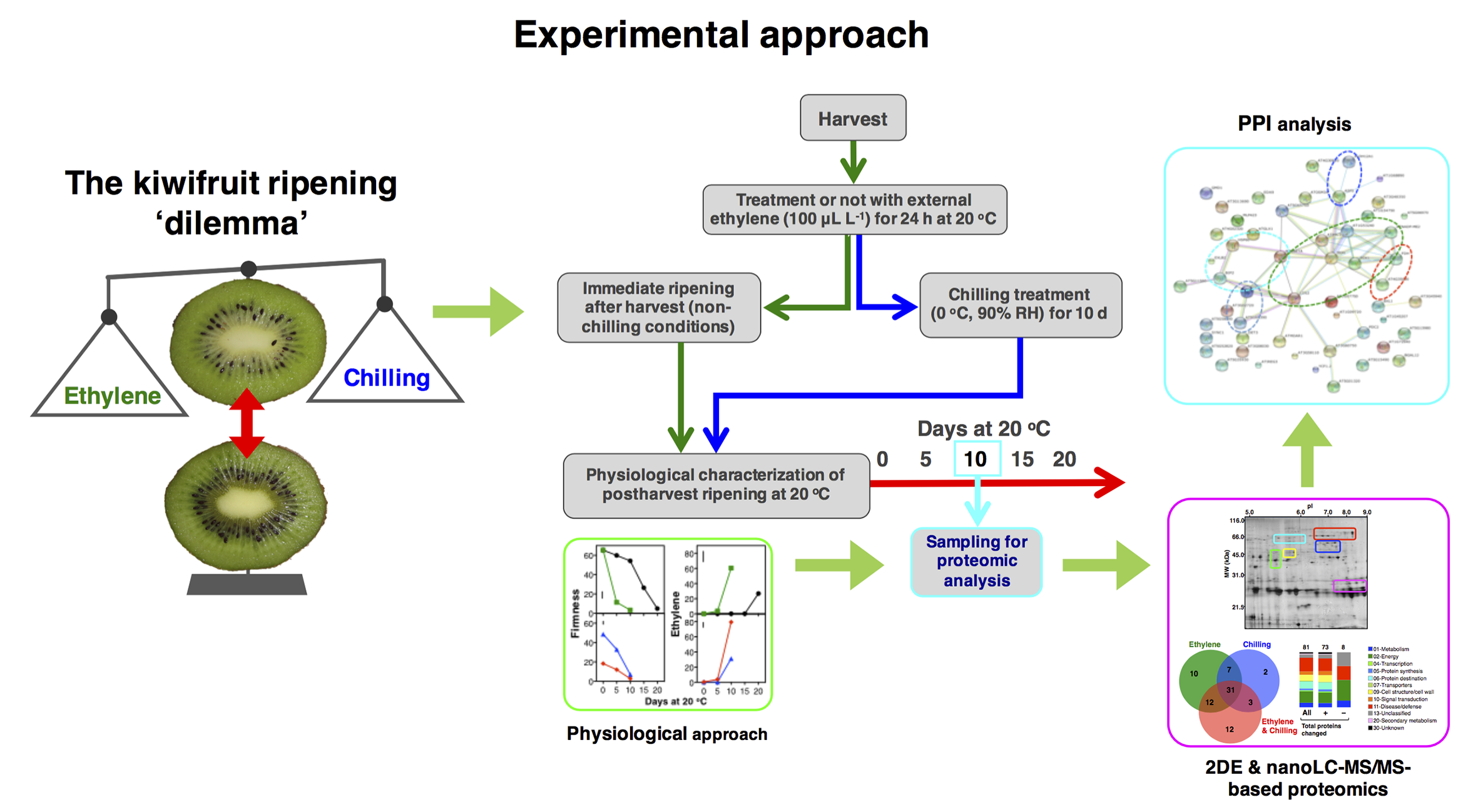

Supplement: Supplementary Figure S1 — Experimental design. [file Image1.TIFF]
